# Metabolic reprogramming of proinflammatory macrophages by target delivered roburic acid effectively ameliorates rheumatoid arthritis symptoms

**DOI:** 10.1038/s41392-023-01499-0

**Published:** 2023-07-28

**Authors:** Na Jia, Yunzhen Gao, Min Li, Yi Liang, Yuwen Li, Yunzhu Lin, Shiqi Huang, Qing Lin, Xun Sun, Qin He, Yuqin Yao, Ben Zhang, Zhirong Zhang, Ling Zhang

**Affiliations:** 1 Key Laboratory of Drug Targeting and Drug Delivery Systems, Ministry of Education, West China School of Pharmacy, College of Polymer Science and Engineering, State key Laboratory of Polymer Materials Engineering, West China School of Public Health and West China Fourth Hospital, Sichuan University, Chengdu, 610041 P. R. China; 2 Department of Pharmacy, West China Hospital, Sichuan University, Chengdu, 610041 China

**Keywords:** Drug development, Rheumatic diseases

## Abstract

Rheumatoid arthritis (RA) is a common chronic inflammatory disorder that usually affects joints. It was found that roburic acid (RBA), an ingredient from anti-RA herb *Gentiana macrophylla* Pall., displayed strong anti-inflammatory activity. However, its medical application is limited by its hydrophobicity, lack of targeting capability and unclear functional mechanism. Here, we constructed a pH responsive dual-target drug delivery system hitchhiking RBA (RBA-NPs) that targeted both CD44 and folate receptors, and investigated its pharmacological mechanism. In rat RA model, the nanocarriers effectively delivered RBA to inflammatory sites and significantly enhanced the therapeutic outcomes compared with free RBA, as well as strongly reducing inflammatory cytokine levels and promoting tissue repair. Following analysis revealed that M1 macrophages in the joints were reprogrammed to M2 phenotype by RBA. Since the balance of pro- and anti-inflammatory macrophages play important roles in maintaining immune homeostasis and preventing excessive inflammation in RA, this reprogramming is likely responsible for the anti-RA effect. Furthermore, we revealed that RBA-NPs drove M1-to-M2 phenotypic switch by down-regulating the glycolysis level via blocking ERK/HIF-1α/GLUT1 pathway. Thus, our work not only developed a targeting delivery system that remarkably improved the anti-RA efficiency of RBA, but also identified a potential molecular target to reversely reprogram macrophages though energy metabolism regulation.

## Introduction

Rheumatoid arthritis (RA) is a common chronic autoimmune disorder which has a complex pathological progression marked by synovial inflammation and joint lesion.^1^ Unfortunately, despite recent advances in immuno-targeted therapies, ~40% RA patients did not respond to treatment using single agent while 5–20% are resistant to all current medications.^2–8^ Therefore, new molecular target and anti-RA agents which provide alternative options are highly demanded. Natural products may provide opportunities for these challenges. It was discovered that roburic acid (RBA) from the extracts of *Gentiana macrophylla* Pall., a herb for treating RA in Southeast Asia, have strong biological activities such as anti-osteoarthritis, anti-inflammatory, and reliving TNF-related conditions.^9–15^ However, it has not been tested in RA model yet and its functional mechanism is unclear. Here, we found that RBA could ameliorate RA symptoms, and the effects appear to be linked to change of local macrophage subpopulations.

Macrophages are highly diverse phagocytes which play important immune roles.^16^ Roughly, the naïve macrophages (M0) can polarize into the classical pro-inflammatory (M1) or immunosuppressive (M2) macrophages after receiving different stimulations.^17–19^ Unsurprisingly, there is an abnormal increase of M1/M2 ratio during the development of RA.^20,21^ Reprogramming M1 macrophages to M2 using targeted IL-10 gene therapy could prevent arthritis-associated joint inflammation and damage.^22^ Thus, manipulating subtype of joint-associated macrophages has considerable therapeutic potential, and makes the hypothesis that RBA reprogramed M1 macrophages more plausible. We thus investigated how RBA rebalanced the macrophage subpopulations. Macrophage polarization and function is closely linked to their metabolic pattern.^23^ Generally, M1 macrophages utilize the glycolytic pathway to meet their high energy demands for pro-inflammatory response;^24^ whereas M2 macrophages predominantly rely on fatty acid oxidation (FAO) and oxidative phosphorylation (OXPHOS) pathways.^25^ During RA progression, macrophages in inflamed joints appear to switch to a hypermetabolic glycolysis state with increasing M1/M2 ratios.^26^ Using glycolytic inhibitor 2-DG or knocking down critical glycolytic enzyme could repress pro-inflammatory response of macrophages.^27^ Thus, it appears that macrophages could be reprogrammed via shifting their metabolic mode. Indeed, we found that RBA could stimulate extracellular regulated protein kinases (ERK), which is a member of the tumor-related mitogen activated protein kinases (MAPKs) family and an upstream activator of hypoxia inducible factor-1α (HIF-1α).^28–30^ HIF-1α is a key component of the hypoxia-responsive master regulator HIF-1, which enhances the expression of proteins involved in glycolysis pathways and shifts energy production mode when activated.^31–33^

Therefore, efficiency of RBA could be enhanced by delivery systems that target M1 macrophage. Motif targeting is a common strategy in nanocarrier design for improved site-specific drug accumulation. CD44 receptor and folate receptor are overexpressed on the surface of pro-inflammatory macrophages in RA synovial.^34,35^ Hyaluronic acid (HA) and folate, which are the natural ligands for CD44 and folate receptors, thus could be used for targeting.^36–38^ Besides, the inflamed joint cavity usually has a lower pH and pH-sensitive delivery system may further improve delivery efficiently.^39^ Hence, we generated a pH-sensitive and CD44/folate dual targeting micelle delivery system based on poly β-amino ester (PAE) to deliver RBA to M1 macrophages in RA joint (RBA-NPs), which significantly improved the therapeutic efficiency of RBA.^40^

In summary, we constructed a self-assembled micelle based on amphiphilic copolymer for RBA delivery (RBA-NPs), and investigated its therapeutic effects and mechanism in RA therapy. The micelles are pH-sensitive and could target M1 macrophages with active dual receptor targeting. As a result, RBA-NPs displayed excellent therapeutic effects in RA model rats, as well as good biosafety. Mechanistic study revealed that RBA could block HIF-1α-mediated glycolysis pathways, which leads to M1-to-M2 macrophage reprogramming, thereby alleviating inflammation and remodeling joint tissues. This work developed an effective RA therapeutic agent based on RBA, discovered the functional mechanism of RBA in RA therapy for the first time, and showed that the ERK/HIF-1α/GLUT1 pathway could be a promising RA treatment target.

## Results

### Preparation and characterization of NPs and RBA-NPs

**Fig. 1:**
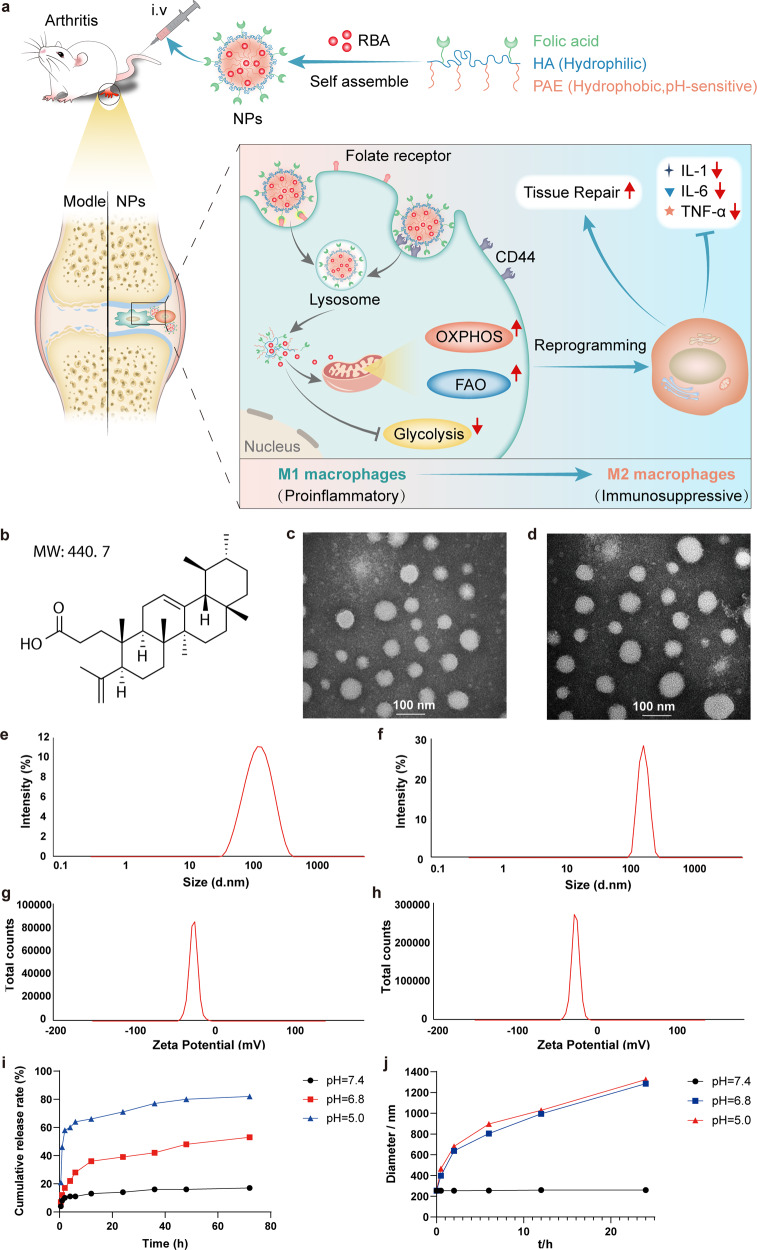
Preparation and characterization of blank NPs and RBA-NPs. **a** Schematic illustration of the preparation and therapeutic mechanism of RBA-NPs against RA. **b** The chemical structure and molecular weight of RBA. **c**, **d** TEM images showed that the blank NPs and RBA-NPs were roughly spherical with uniformed size. **e**, **f** The particle sizes of blank NPs and RBA-NPs were about 174.2 nm and 249.4 nm as measured by DLS. **g**, **h** The zeta potentials of blank NPs and RBA-NPs were −19.9 mV and −17.6 mV, respectively. **i** The in vitro release of RBA from RBA-NPs at different conditions (pH = 5.0, 6.8 or 7.4) in 72 h. **j** Stability of RBA-NPs at different conditions (pH = 5.0, 6.8 or 7.4) in 24 h

PAE-HA-FA copolymer was synthesized mainly by Michael-addition polymerization and amidate reaction (Supplementary Fig. 1). PAE-HA-FA appeared white translucent massive solid with a melting point of 160 ~ 161 °C, molecular weight of 12000 and yield of 89%. The chemical structures of the obtained product PAE-HA-FA graft copolymer was confirmed by FT-IR (Supplementary Fig. 2), ^1^H-NMR spectroscopy and ^13^C-NMR spectroscopy (Supplementary Fig. 3). The fabricated copolymer could self-assemble into nanomicelle for targeted delivery of RBA (Fig. 1a, b). RBA-loaded FA-HA-PAE nanoparticles (RBA-NPs) and blank FA-HA-PAE nanoparticles (NPs) were both prepared using ultrasonic method. Transmission electron microscopy (TEM) images showed that the blank NPs and RBA-NPs were roughly spherical with uniformed size (Fig. 1c, d). The particle sizes of blank NPs and RBA-NPs were about 174.2 nm and 249.4 nm as measured by DLS (Fig. 1e, f); and the zeta potentials of blank NPs and RBA-NPs were −19.9 mV and −17.6 mV, respectively (Fig. 1g, h). In addition, the critical micelle concentration (CMC) and serum stability tested indicates that the micelles are generally stable in physiological conditions (Supplementary Fig. 4a, b). FA-HA-PAE and RBA in vitro analysis methods were established in Supporting methods, Supplementary Table 1–4, Supplementary Fig. 5, 6. The purity of FA-HA-PAE was 97.29% which can be used for subsequent experiments. The encapsulation efficiency (EE) and drug loading (DL) of RBA-NPs was 84.2 ± 1.3% and 9.6 ± 0.8%, respectively.

Because the PAE core is designed to degrade at low pH, we examined the cumulative release rates of RBA from polymeric micelles and the particle size of blank micelles in different pH conditions in vitro. PBS buffer at pH 7.4, 6.8 and 5.0 was used to imitate the normal physiological condition, microenvironment of RA joints and cyto-lysosome situation, respectively.^41^ As shown in Fig. 1i, at pH 7.4, the RBA-NPs were relatively stable and the release rates of RBA were slow, (~14% RBA released at 24 h and ~17% at 72 h) without apparent burst release. In comparison, at pH 6.8, ~50% RBA was released at 24 h and ~59% at 72 h, and the leakage of RBA was further accelerated at pH 5.0, reaching ~71% at 24 h and ~82 % at 72 h. Consistently, the nano micelles were stable after 24 h incubation at pH 7.4, and the micelle size increased significantly at pH 6.8 and 5.0 (Fig. 1j). Thus, the RBA-loading nanomicelles were successfully generated with designated pH sensitivity.

### RBA-NPs display targeting capability both in vitro and in vivo

**Fig. 2:**
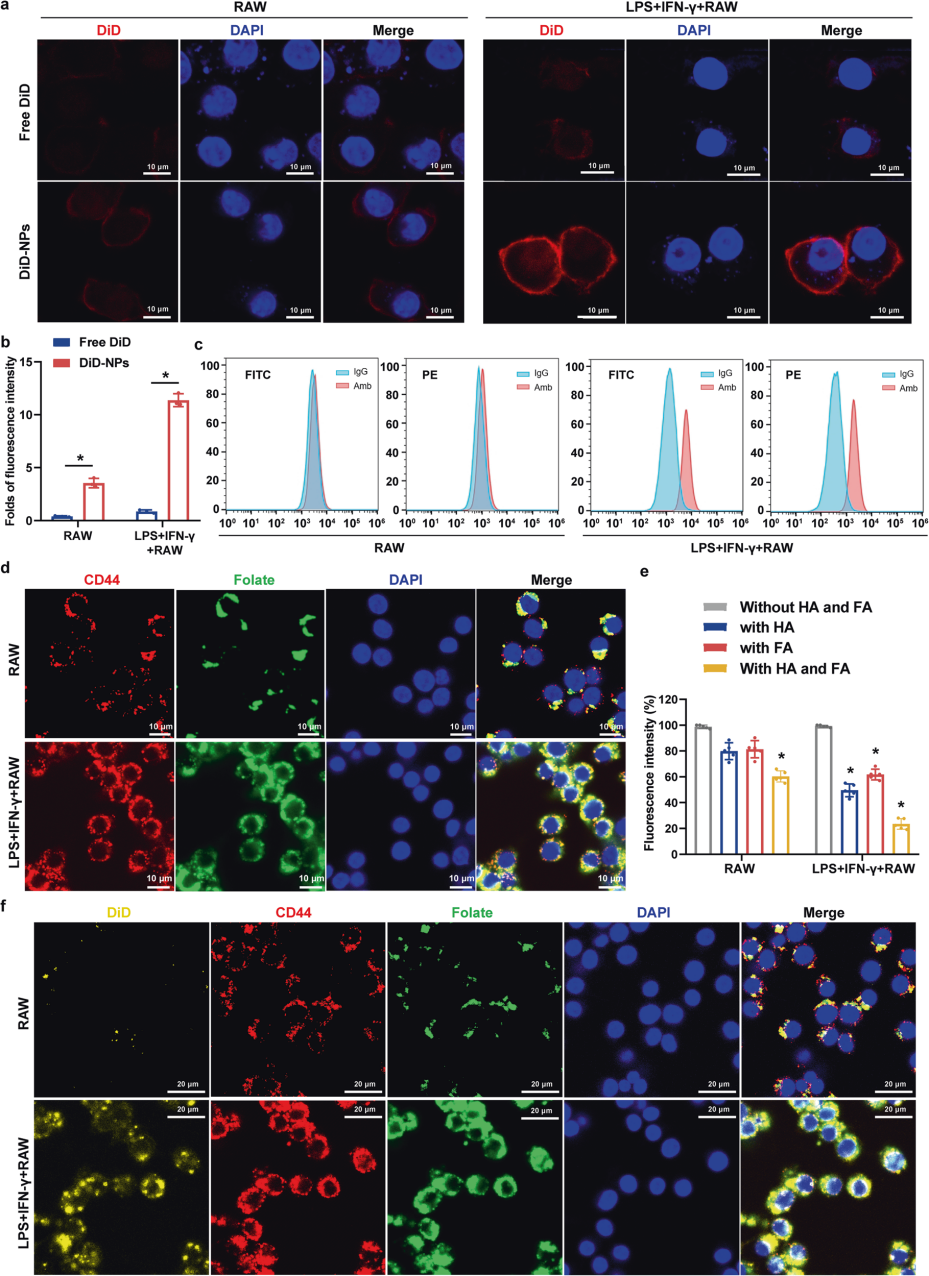
RBA-NPs display targeting capability in vitro. **a**, **b** Confocal micrographs showed that DiD-NPs (red) enjoyed significantly improved cell uptake compared with free DiD in LPS + IFN-γ activated RAW264.7 cells. Scale bar = 10 μm. **c** Flow cytometry analysis showed that the fluorescence intensity of DiD-NPs in LPS + IFN-γ activated RAW264.7 cells was higher than that of free DiD in RAW264.7. **d** The expression of CD44 and folate receptors increased significantly on the surface of LPS + IFN-γ activated RAW264.7 cells (**d**). Scale bar = 10 μm. **e** LPS + IFN-γ activated macrophages were pretreated with HA or FA or both HA and FA to compete for the overexpressed CD44 or folate receptors, and cellular uptake of DiD-NPs was reduced. **f** Confocal micrographs showed co-localization of DiD-NPs (orange) with CD44 receptor (red) and folate receptor (green). Scale bar = 20 μm. Cell nuclei was stained with DAPI (blue). All results are shown as mean ± SD. *P < 0.05. The dose of LPS and IFN-γ was 100 ng/mL and 20 ng/mL

The targeting capability of fabricated nanocarriers was then examined. Here, 1,1'-Dioctadecyl-3,3,3',3'-Tetramethylindodicarbocyanine (DiD) was used as a marker instead of RBA (DiD-NPs), to label the uptake of nano-micelles in cells and the fluorescence localization in animals.^42^ First, cellular uptakes of free DiD and DiD-NPs were assessed by CLSM and flow cytometry in RAW264.7 and LPS + IFN-γ activated RAW264.7 cell in vitro. CLSM results showed that DiD-NPs enjoyed significantly improved cell uptake compared with free DiD (Fig. 2a, b). Flow cytometry analysis showed that the fluorescence intensity of DiD-NPs in LPS + IFN-γ activated RAW264.7 cells was about 4.98 times higher than that of free DiD, and 1.33 times in RAW264.7 (Fig. 2c and Supplementary Fig. 7). The CD44 and folate receptor targeting dependency of the fabricated NPs was then verified. The expression of CD44 and folate receptors increased significantly on the surface of LPS + IFN-γ activated RAW264.7 cells (Fig. 2d). When LPS + IFN-γ activated RAW264.7 cells were pretreated with free HA or FA to compete for the overexpressed CD44 or folate receptors, the uptake of DiD-NPs was reduced to 51.8% and 65.1% of untreated cells respectively; and when both HA and FA were added, the signal level dropped to 24.5% (Fig. 2e). Confocal micrographs show that DiD-NPs colocalized with CD44 receptor and folate receptor, indicating that DiD-NPs could target dual receptors on the surface of proinflammatory macrophages (Fig. 2f).

**Fig. 3:**
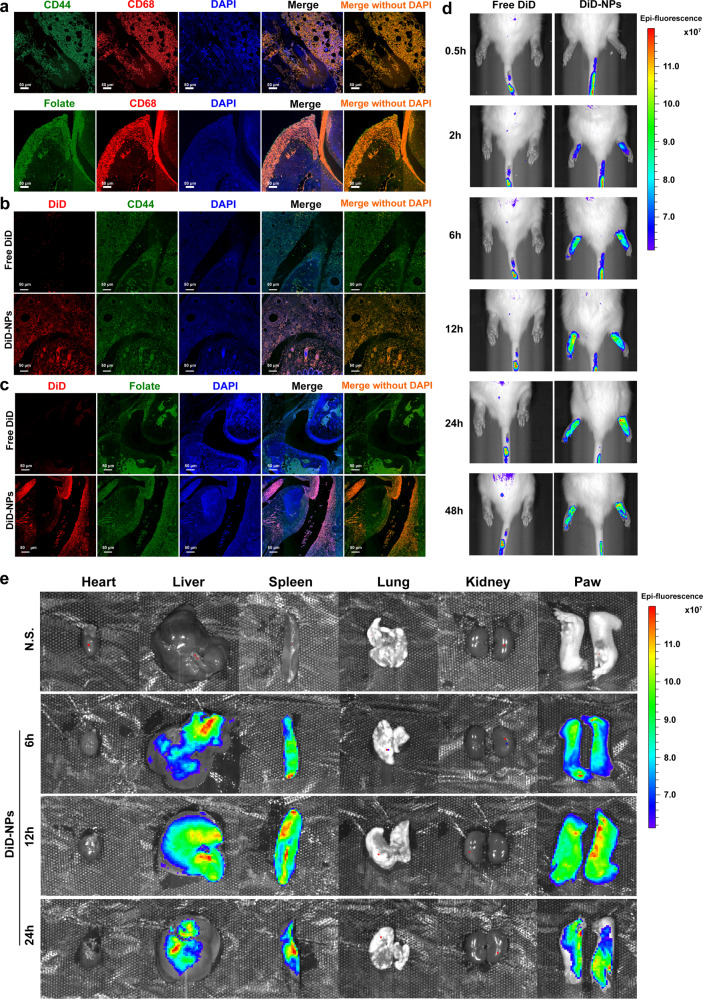
RBA-NPs display targeting capability in vivo. **a** Confocal micrographs showed that CD44 receptor (green) and folate receptor (green) expression levels on macrophages in the inflamed joints of AIA rats. Macrophages were marked by CD68 antibody (red). Cell nuclei were stained with DAPI (blue). Scale bar = 50 μm. **b**, **c** Confocal micrographs showed co-localization of free DiD and DiD-NPs with CD44 receptor and folate receptor in the inflamed joints of AIA rats. Free DiD and DiD-NPs were colored red. CD44 receptor and folate receptor were colored green. Cell nuclei were stained with DAPI (blue). Scale bar = 50 μm. *n* = 5 independent animals. **d** The imaging of DiD fluorescence in joints after intravenous administrated with free DiD or DiD-NPs at different time points after administration. *n* = 5 independent animals. The DiD-NPs group showed higher fluorescence intensity at each time point, and fluorescence persisted as long as 48 h. **e** The distribution of DiD-NPs in heart, liver, spleen, lung, kidney, and joints in AIA rats were evaluated at 6, 12 and 24 h after administration. *n* = 5 independent animals. The fluorescence intensities were stronger in inflamed joints

Then, the biodistribution of NPs was investigated in adjuvant-induced arthritis (AIA) rat model which was established by subcutaneous injection with CFA into the base of rat tails. The modeled rats were then intravenously injected with free DiD or DiD-NPs via the tail vein. The arthritic joints were sectioned and stained by immunofluorescent methods. Again, it can be seen that CD44 receptor and folate receptor were overexpressed on inflammatory macrophages (as marked by CD68) in inflamed joints (Fig. 3a). Consistent with in vitro data, the DiD fluorescence in the synovial joint of DiD-NPs treated group is higher than free DiD group (Fig. 3b, c). Furthermore, the DiD signal of DiD-NPs group is mainly overlapped with CD44 receptor and folate receptor whereas free DiD group showed low levels of colocalization of CD44 receptor and folate receptor. Consequently, fluorescence intensities of DiD in joints at 0.5, 2, 6, 12, 24 and 48 h after administration were measured using an in vivo imaging system (Fig. 3d and Supplementary Fig. 8a). Fluorescence signal was negligible in the inflamed joints of AIA rats treated with free DiD, while intense fluorescence signals in arthritic joints were observed as early as 0.5 h in the NPs groups. The DiD-NPs group showed higher fluorescence intensity at each time point, and fluorescence persisted as long as 48 h. The distribution of the DiD-NPs in heart, liver, spleen, lung, kidney, and joints of AIA rats were also evaluated at 6, 12 and 24 h after administration (Fig. 3e and Supplementary Fig. 8b). It is clear that the fluorescence intensities were stronger in inflamed joints than in heart, lung, and kidney. The high fluorescent level in spleen may related to the fact that spleen is a major immune organ. These results together show that the fabricated dual-targeting NPs could efficiently and selectively deliver loaded cargoes to proinflammatory macrophages and achieve remarkably enhanced accumulation in inflamed joints of AIA rats.

### RBA-NPs inhibit inflammation and promote bone erosion repair in AIA rats

**Fig. 4:**
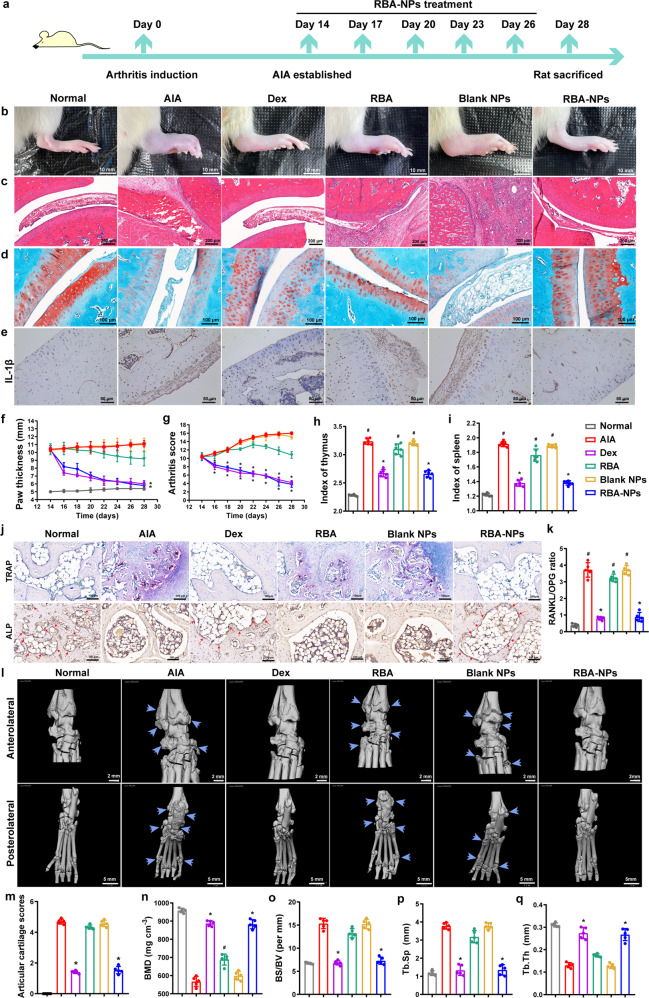
RBA-NPs inhibit inflammation and promote bone erosion repair in AIA rats. **a** The schematic illustration of RBA-NPs treatment. **b** Representative photographs of hindlimbs at the endpoint of the experiment from different treatment groups. Scale bar = 10 mm. **c** Histopathology evaluation of ankle joints was identified using H&E. Scale bar = 100 μm. **d** Detection of cartilage injury of rats ankle joint in each group by Safranin O-Fast green staining (*n* = 5 independent animals). Scale bar = 100 μm. **e** Immunohistochemical analyses of of IL-1β expression levels in arthritic joints in different groups (*n* = 5 independent animals). Scale bar = 80 μm. IL-6 and TNF-α expression levels in arthritic joints were showed in Supplementary Fig. 8. **f**, **g** Paw thickness and arthritis score of AIA rats were recorded every other day during the treatment period. Data represent mean ± SD (*n* = 7 independent animals). *P < 0.05 vs. N.S. group. **h**, **i** Index of spleen and thymus of AIA rats were recorded every other day during the treatment period. Data represent mean ± SD (*n* = 7 independent animals). ^#^*P* < 0.05 vs. Normal group; *P < 0.05 vs. N.S. group. **j** Detection of TRAP-stained osteoclast and ALP-stained osteoblast expression levels in arthritic joints in different groups (*n* = 5 independent animals). Scale bar = 100 μm. Osteoclasts located in the marrow cavity were stained red. TRAP-positive multinuclear cells that contained more than three nuclei were denoted as osteoclasts. **k** RANKL/OPG ratio in arthritic joints from rats receiving the indicated treatment. Data represent mean ± SD (*n* = 5 independent animals). **l** Representative micro-CT images of the anterolateral and the posterolateral ankle joints at the endpoint of the experiment from different treatment groups in therapeutic efficacy study (*n* = 6). Scale bar = 2 mm. **m**–**q** Quantitative micro-CT analysis of articular cartilage scores, BMD, BS/BV, Tb.Sp and Tb.Th of the ankle joints at the endpoint of the experiment. Data represent mean ± SD (n = 3 independent animals)

Next, the therapeutic efficacy of RBA-NPs treatment was evaluated in AIA rats (Fig. 4a). In this part, a glucocorticoid drug dexamethasone (Dex) was selected as the positive drug control group. Dex is currently a common drug for alleviating the symptoms of RA patients due to its powerful anti-inflammatory and analgesic effects. The administration scheme of Dex group was the same as that of RBA-NPs group. After 14 days of arthritis induction, the ankles and paws of AIA rats showed severe swelling. Saline or RBA-NPs were intravenously injected into rats (5 mg/kg RBA). As illustrated in Fig. 4b, f and g, RBA-NPs and Dex showed strong therapeutic efficacy, significantly relieving disease development and reducing paw swelling after administration. In contrast, paw thickness and arthritis score of AIA rats in blank NPs group barely differed with AIA group, while free RBA could only slightly reduce paw swelling and arthritis score. To further evaluate joint inflammation and cartilage destruction levels, histological analysis of rat ankle joints slices was conducted. H&E-stained sections in AIA and blank NPs group showed a large number of inflammatory cell infiltration and severe synovial hyperplasia. Compared with AIA group, free RBA group had limited effect in relieving these symptoms, while RBA-NPs and Dex strongly reduced synovial inflammation (Fig. 4c). Safranin-O Fast-Green staining revealed that most of cartilage and bone tissues disappeared in some joint sections in AIA and blank NPs group. In sharp contrast, intact and red-stained cartilage could be found in joints of RBA-NPs and Dex groups, demonstrating that RBA-NPs effectively lessened articular cartilage lesion (Fig. 4d). Immunologically, the spleen and thymus indices were significantly increased in the AIA group compared to normal rats, which were markedly reduced in RBA-NPs and Dex treated group to almost the control level (Fig. 4h, i). The immunohistochemical assays revealed that RBA-NPs effectively down-regulated the secretion of inflammatory cytokines such as IL-1β (Fig. 4e), IL-6 (Supplementary Fig. 9) and TNF-α (Supplementary Fig. 9) in ankle joints.

To check whether RBA-NPs could restore bone function and promote tissue repair, we evaluated the effects of RBA-NPs on the expression levels of related markers using tartrate-resistant acid phosphatase (TRAP)-stained osteoclast and alkaline phosphatase (ALP) immunohistochemical-stained osteoblast in arthritic joints (Fig. 4j). Osteoclasts located in the marrow cavity were stained red when using TRAP staining assay, and TRAP-positive multinuclear cells that contained more than three nuclei were denoted as osteoclasts. Results showed that rats in AIA group suffered heavy osteoclasts and bone erosion in ankle joints, while RBA-NPs and Dex reduced number of osteoclasts and promoted bone damage repair, with significantly increased ALP expression (Arrows were used to mark the places where osteoblast produced). Further experiments showed that RBA-NPs and Dex lowered the expression levels of receptor activator of nuclear factor-κB ligand (RANKL) and up-regulated osteoprotegerin (OPG) level in rat arthritic joints, leading to a reduced RANKL/OPG ratio (Fig. 4k and Supplementary Fig. 10).

The bone erosion was then further verified in a more visualized way using micro-CT analysis (Fig. 4l). The top and bottom images represented the anterolateral and posterolateral ankle joints in the same rat. Arrows were used to mark the places where bone tissue damage occurred. The AIA and blank NPs groups showed a rough bone surface and severe bone erosion in the inflamed ankle at day 28 after treatment. There was also a significant reduction in bone mineral density (BMD), an increase in articular cartilage scores and BS/BV in these two groups compared with the normal rats. While free RBA only provided moderate relief on bone erosion, the RBA-NPs and Dex treatment resulted in smooth bone surface and high BMD close to that of the normal rats (Fig. 4m–o). Unsurprisingly, RBA-NPs and Dex treatment showed the most prominently effects in reducing trabecular separation (Tb.Sp) and increasing trabecular bone thickness (Tb.Th) (Fig. 4p, q). All these data support that RBA-NPs could effectively suppress the progression of cartilage injury and promote bone erosion repair in AIA rats.

### RBA-NPs reprogram proinflammatory M1 macrophages to anti-inflammatory M2 macrophages

As reported, rebalancing the inadequate M1/M2 ratio in RA joints could mitigate joint damage as M1 appears to be able to drive the pathological process of RA.^43^ Since RBA-NPs were efficiently internalized by M1 macrophages and significantly inhibited inflammation and tissue damage, it is necessary to explore its role in M1/M2 polarization.

**Fig. 5:**
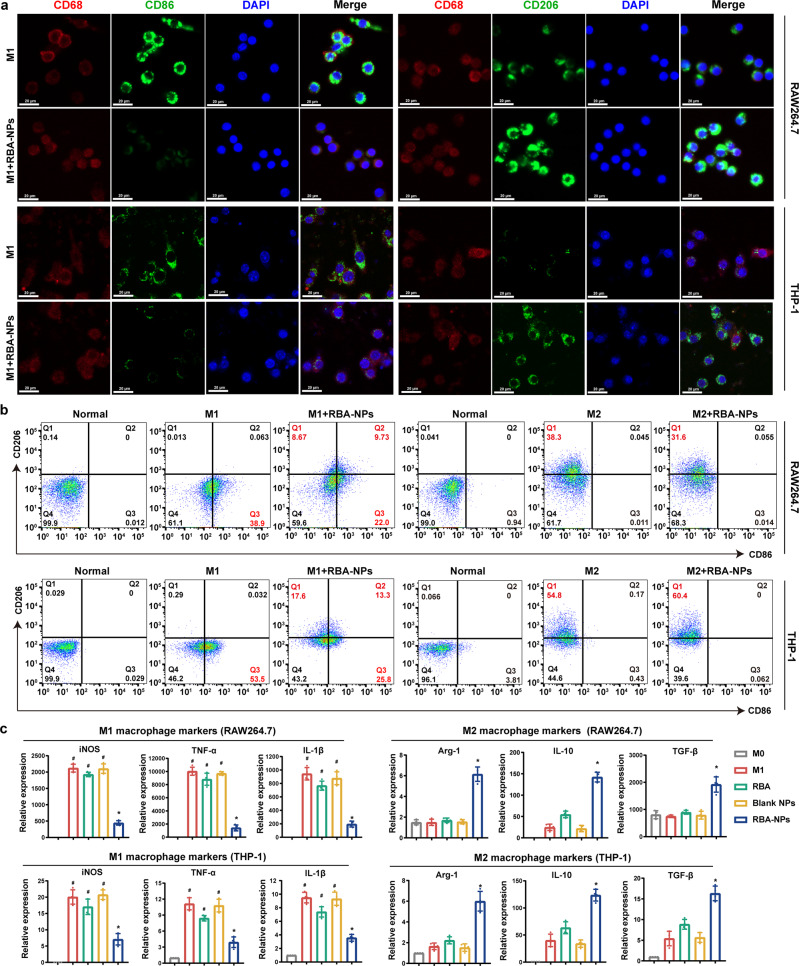
RBA-NPs reprogram proinflammatory M1 macrophages to anti-inflammatory M2 macrophages in RAW264.7 and THP-1 cells. **a** RAW264.7 and THP-1 cells were pretreated by LPS + IFN-γ or IL-4 + IL-13 to induce M1 or M2 phenotype polarization. Immunofluorescence staining of CD68 (red, pan-macrophage markers), CD86 (M1 marker, green) or CD206 (M2 marker, green), and nuclei (blue) on M1 phenotype macrophages without or with treatment RBA-NPs. Scale bar = 20 μm. **b** The proportions of M1 phenotype macrophages (CD86+) and M2 phenotype macrophages (CD206+) without or with treatment RBA-NPs were detected by flow cytometry assay in RAW264.7 and THP-1 cells. **c** The expressions of M1 phenotype (iNOS, TNF-α, IL-1β) and M2 phenotype (Arg-1, IL-10, TGF-β) macrophage makers genes were detected in RAW264.7 and THP-1 cells. Data were expressed as mean ± SD, *n* = 5. ^#^*P* < 0.05 vs. Normal group; *P < 0.05 vs. M1 group

To check this possibility, mouse and human macrophage cell lines RAW264.7 and THP-1 cells were used. These cells were pretreated by LPS + IFN-γ or IL-4 + IL-13 to induce M1 or M2 phenotype polarization. These cells were then analyzed by immunofluorescence staining of CD68 and F4/80 (the pan-macrophage markers), CD86 (M1 marker), and CD206 (M2 marker). As expected, a notable increase in CD86 was found in LPS and IFN-γ pretreated RAW264.7. After RBA-NPs treatment, the fluorescence intensity of CD86 decreased while the fluorescence intensity of CD206 increased, suggesting that macrophages were possibly repolarized from M1 to M2 macrophages (Fig. 5a and Supplementary Figs. 11–13). On the other hand, there is no noticeable change on M2 macrophages after RBA-NPs treatment. THP-1 cells gave similar results (Fig. 5a and Supplementary Fig. 14). These data thus suggested that RBA-NPs effectively promoted M1-to-M2 repolarization of macrophages in both mouse and human macrophages.

Moreover, this hypothesis was also verified by flow cytometry using RAW264.7 and THP-1 cells (Fig. 5b, Supplementary Fig. 15, 16). The ratio of M1 and M2 macrophages shifted from 38.9% and 0.076% to 29.7% and 18.4% in RAW264.7 cells after RBA-NPs treatment, respectively. In THP-1 cells, the ratio changed from 53.5% and 0.32% to 25.8% and 30.9%, respectively. Finally, RBA-NPs prominently suppressed the expression levels of M1 markers including iNOS, TNF-α, IL-1β and raised levels of M2 markers including Arg-1, IL-10, and TGF-β (Fig. 5c) in both cell lines. Collectively, we conclude that RBA-NPs caused the M1 to M2 phenotypic transition of macrophages.

### RBA-NPs drive M1-to-M2 phenotypic switch by down-regulating the glycolysis activity level

**Fig. 6:**
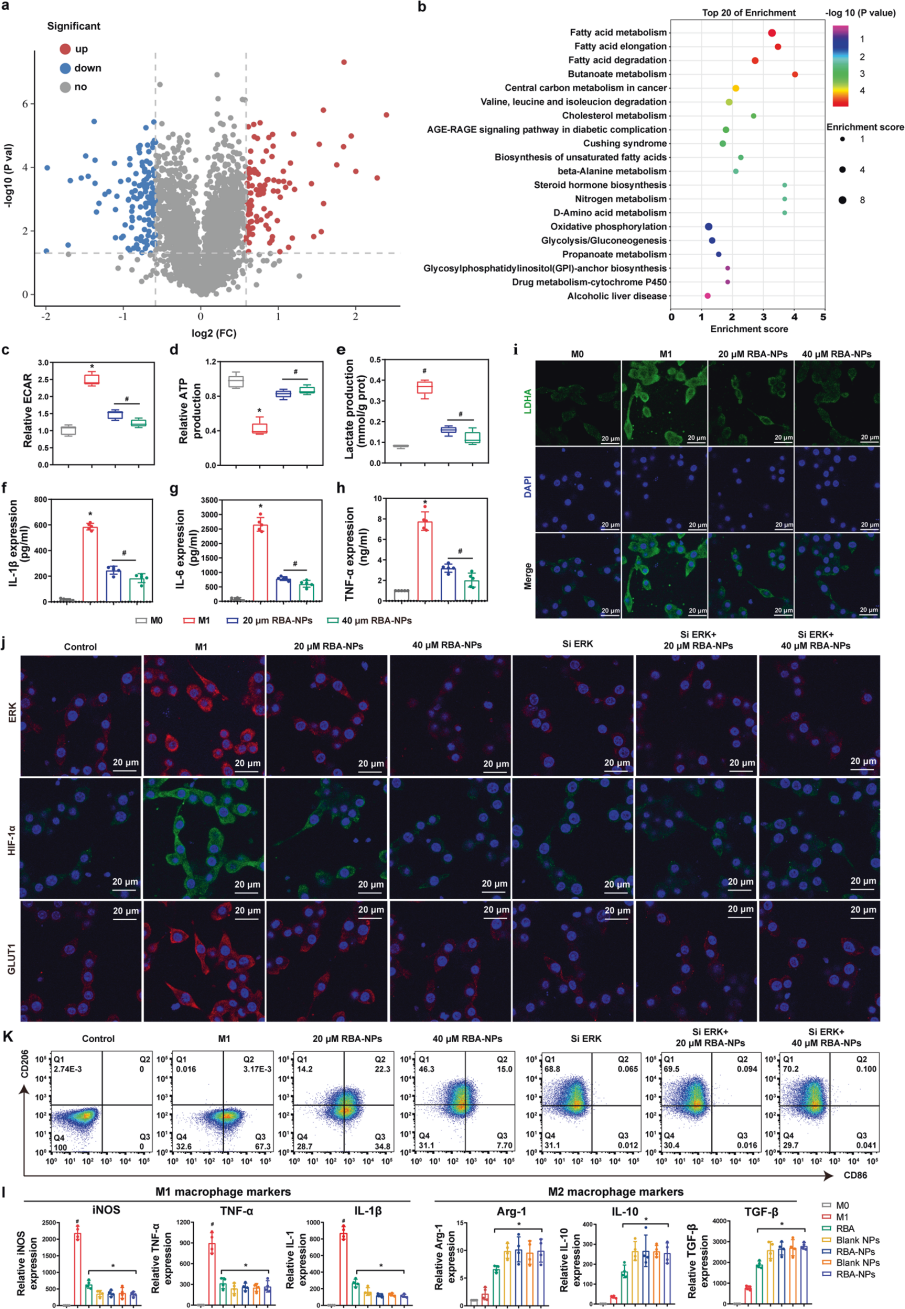
RBA-NPs drive M1-to-M2 phenotypic switch by down-regulating the glycolysis level via blocking ERK/HIF-1α/GLUT1 pathway. **a** Volcano plot of all proteins identified in this study. Red and blue dots indicate up-or down-regulated proteins significantly, respectively (folds change of >1.5 or <0.667 and *p*-value of <0.05). **b** Significantly changed pathways of the differentially expressed up-regulated genes based on the KEGG analysis. The proteins affected by RBA-NPs treatment were enriched in fatty acid metabolism, glycolysis and other pathways. **c**–**e** The histogram of ECAR, relative ATP, lactate levels in different groups. The production of ECAR, relative ATP, lactate could reflect the glycolysis ability. Data represent mean ± SD (*n* = 5). *P < 0.05 vs. M0 group; ^#^*P* < 0.05 vs. M1 group. **f**–**h** IL-1β, TNF-α, and IL-6 levels were analyzed by the kits. Data represent mean ± SD (*n* = 5). *P < 0.05 vs. M0 group; ^#^*P* < 0.05 vs. M1 group. **i** Immunofluorescence staining of LDHA (green) and nuclei (blue) on M1 macrophages in different groups. Scale bar = 20 μm. LDHA was the last step in the process of catalytic glycolysis and an important indicator for measuring glycolysis process. **j** Immunofluorescence staining of ERK (red), HIF-1α (green), GLUT1 (red) and nuclei (blue) on M1 macrophages in different groups. Scale bar = 20 μm. RBA-NPs significantly blocked ERK/HIF-1α/GLUT1 pathway. **k** The proportions of M1 phenotype macrophages (CD86 + ) and M2 phenotype macrophages (CD206 + ) on M1 macrophages in different groups without or with treatment siERK were detected by flow cytometry assay. **l** The expressions of M1 phenotype (iNOS, TNF-α, IL-1β) and M2 phenotype (Arg-1, IL-10, TGF-β) macrophage makers genes were detected. Data were expressed as mean ± SD, *n* = 4. ^#^*P* < 0.05 vs. Normal group; *P < 0.05 vs. M1 group

We then explored the mechanisms of macrophages reprogramming by RBA-NPs using proteomics methods. In total, 4068 proteins were identified in analyzed macrophages (Supplementary Fig. 17a). Compared with control, RBA-NPs treatment significantly changed the expression levels of 235 proteins, of which 106 were up-regulated and 129 were down-regulated (Fig. 6a, Supplementary Fig. 17b, Supplementary materials 1). The functions of these significantly affect proteins were annotated using Gene Ontology (GO) and Kyoto Encyclopedia of Genes and Genomes (KEGG) enrichment analysis. The GO analysis results indicated that these proteins were mainly involved in metabolic process, immune system process, innate and adaptive immune response, inflammatory response, and catalytic activity (Supplementary Fig. 18a–c, Supplementary Fig. 19a–d). It is unsurprising that immune and inflammatory proteins were involved, but the high number of metabolic related proteins identified was unexpected. Thus, related metabolomics pathways of these proteins were further analyzed by KEGG enrichment (Fig. 6b). It can be seen that proteins affected by RBA-NPs treatment are enriched in fatty acid metabolism, glycolysis and other pathways. Hence, RBA-NPs treatment appeared to adjusted the energy metabolism and immune system at the protein translation levels. As introduced, the immune microenvironment of RA promoted metabolic reprogramming from FAO to glycolysis in macrophages.^26^ Interestingly, the characteristic metabolic modes of M1 and M2 macrophage were consistent with the results of proteomics. We then used STRING database to construct protein-protein interaction (PPI) networks to analyze the functional relationships of metabolic relative proteins (Supplementary Fig. 20 and Supplementary materials 2). The PPI networks again show that the most concentrated proteins in nodes were those involved in three important metabolic processes: glycolysis, FAO and OXPHOS, where glycolysis is often considered a major metabolic pattern.

Thus, glycolytic capacity in M1 macrophages was examined to check the regulation of intracellular energy metabolism by RBA-NPs. The extracellular acidification rate (ECAR) is an indicator of glycolysis activity.^44^ Results show that the ECAR in M1 macrophages was 2.5-fold higher than normal cells (Fig. 6c). After 20 or 40 μM RBA-NPs treatment, the ECAR of M1 macrophages nearly returned to normal. The ATP production efficiency is another indicator of glycolysis as the ATP production efficiency of glycolysis is far lower than FAO.^45^ As shown in Fig. 6d, the low ATP production of M1 macrophages could be raised by RBA-NPs treatment, where higher dose gave stronger effect. RBA-NPs also decreased lactate production in a dose-dependent manner, which is an important product during glycolysis (Fig. 6e). At the same time, RBA-NPs suppressed the high level of lactate dehydrogenase A (LDHA) (Fig. 6i), which plays key role in the last step of catalytic glycolysis, in M1 macrophage.^46^ In addition, RBA-NPs inhibited the expressions of IL-1β, IL-6 and TNF-α in M1 macrophages (Fig. 6f–h). These data all suggest that RBA-NPs down-regulated the glycolysis level in M1 macrophages, and the higher dose leads to stronger effect.

### RBA-NPs drive M1-to-M2 phenotypic switch by the blocking the ERK/HIF-1α/GLUT1 pathway

In inflammatory state, HIF-1α could enhance the synthesis of lactate and up-regulate target genes such as LDHA and GLUT1, and the elevated expression of HIF-1α is a key feature of glycolysis of M1 macrophages.^47^ In early investigations, we found that the increased expression of HIF-1α was markedly suppressed by RBA-NPs in M1 macrophages and the HIF-1α inhibitor PX-478 showed functional redundancy with RBA-NP (Supplementary Figs. 21–23). We then further explored how RBA acts in relation to HIF-1α-related pathways.

First, we collected the RBA-NPs treated and untreated mouse M1 macrophage samples and processed them for proteomics analysis. The KEGG enrichment based on the proteomics data show that four types of proteins in HIF-1 signaling pathway (ko04066) are significantly changed: glucose transporter 1 (GLUT1), mitogen-activated protein kinase (MAPK), FAD-binding FR type domain containing protein (Cyb), and eukaryotic initiation factor 4E2 (EIF4E2) (more details in Supplementary Table 5). GLUT1, which were involved in processes such as glucose metabolism and immune response,^48^ experienced the highest change (p-value: 5.65231E-06). More specifically, GLUT1 functions downstream of HIF-1α, and accelerates glucose transport to support the high levels of glycolysis in proinflammatory macrophages.^49^ The mitogen-activated protein kinases (MAPKs) were the second most affected (p-value: 2.04611E-05). As an important member of MAPK family, ERK is able to regulate the expression of HIF-1α.^50^ Therefore, it appears that the ERK/HIF-1α/GLUT1 pathway is strongly affected by RBA-NPs.

In following experiments, ERK and GLUT1 were further investigated. Compared with static macrophages, the content of ERK, HIF-1α and GLUT1 protein in M1 macrophages were indeed increased (Fig. 6j and Supplementary Fig. 24). Treatment with 20 μM RBA-NPs restrained the level of these proteins, and 40 μM RBA-NPs, resulted in stronger effects, indicating that repression of RBA-NPs on ERK, HIF-1α and GLUT1 was concentration dependent. Then, ERK siRNA was used to knock down ERK (Fig. 6j). Under this condition, RBA-NPs did not affect the expression of HIF-1α and GLUT1, which is consistent with the current signaling model that ERK is upstream of HIF-1α and GLUT1.

Further flow cytometry analysis also showed that RBA-NPs basically had no effect on M1/M2 ratio after ERK knock down, as the depletion of ERK significantly increased the portion of M2 macrophage to as high as 68.8% by itself (Fig. 6k and Supplementary Fig. 25). In contrast, without siERK treatment, RBA-NPs again reduced the portion of CD68-positive macrophages from and elevated number of CD206-positive macrophages. Besides, the expression assay of M1 markers including iNOS, TNF-α, IL-1β and M2 markers including Arg-1, IL-10, TGF-β also gave consistent results (Fig. 6i).

Therefore, these evidences all support that RBA-NPs possibly reprogrammed macrophage polarization via blocking the ERK/HIF-1α/GLUT1 pathway.

### Preliminary safety evaluation of RBA-NPs

The biosafety of RBA-NPs was also briefly evaluated both in vitro and in vivo. Initially, cytotoxicity of NPs was assessed using RAW264.7 and THP-1 cells in vitro. The cells were exposed to NPs at different concentrations for 24 h. The results revealed that cell viability was still above 90% at a concentration as high as 40 μg/mL in both cells (Supplementary Figs. 26, 27). The cytotoxicity of free RBA and RBA-NPs were dose-dependent, with the cell survival rate dropped from 80% at 40 μM dose to 50%. at 100 μM (Supplementary Figs. 28, 29). The results indicate that RBA was the main factor affecting the cell activity, while the delivery vehicle did not affect the cell activity within the concentration range under investigation.

Then, healthy rats were treated by different dosage of RBA-NPs, and blood, urine, and important organs from these rats were sampled after predetermined time length and examined. Liver function indicators alanine aminotransferase (ALT) and aspartate aminotransferase (AST) (Supplementary Fig. 30a, b); heart function indicators creatine kinase (CK) and lactic dehydrogenase (LDH) (Supplementary Fig. 30c, d); kidney function indicators creatinine (CREA) and uric acid (UA) (Supplementary Fig. 30e, f); spleen function indicators white blood cell (WBC), red blood cell (RBC) and platelet (PLT) (Supplementary Fig. 30g–i); lung tissue edema indictor lung W/D weight ratio (Supplementary Fig. 30j); and rat body weight (Supplementary Fig. 30k) were all measured. These indicators are not significantly shifted after RBA-NP treatment.

Finally, hearts, livers, spleens, lung, kidneys samples were sectioned and H&E stained (Supplementary Fig. 31). The images showed that RBA-NPs did not induce clear necrosis or inflammation in these organs. Thus, RBA-NPs appear to be biocompatible and does not cause acute damage to major organs.

## Discussion and conclusion

The M1-M2 macrophage phenotype switch is the central theme in this study. We unexpectedly found that alternating the metabolic pathway of M1 macrophages appeared to reversely regulated their polarization (Fig. 5), as polarization is often assumed to be the cause of metabolic change and subsequent functional alternation (e.g. releasing of inflammatory cytokines).^45,46^ There are only two recent reports providing evidences of such metabolic-driven M1-to-M2 reprogramming as far as we know.^47,48^ However, there are literatures that support this repolarization mechanism in some ways. First, robust glycolysis in macrophages maintains the transcription of inflammatory factors and assist the M1 phenotype polarization, and inhibiting glycolysis could affect their typical functions including phagocytosis, secretion of pro-inflammatory cytokines, and ROS production.^49–51^ Second, M2 macrophage polarization in tumor is also heavily influenced by glutaminolysis and other related metabolic factors.^52–54^

In general, glycolysis regulation signaling pathways appears to be heavily linked to macrophage phenotype. The hypoxia-related HIF-1α pathway seems to play a central role in the increased M1 polarization in RA. In RA affected joint, the metabolic shift from FAO and OXPHOS to glycolysis in macrophages enables energy to be produced independent of oxygen supplys.^55^ HIF-1α binds to HIF-1β to form HIF-1 after nucleus translocation in hypoxic conditions.^47^ HIF-1 then binds to the hypoxia-responsive elements and enhances the transcription of *IL-1β* and genes involved in glycolysis pathways, such as *GLUT1*.^33^ As the glucose transporter, GLUT1 up-regulate glycolytic rates and blunted respiratory capacity.^56^ For instance, gout and pseudo-gout-related crystals led to a metabolic rewiring toward the aerobic glycolysis pathway by an increase in GLUT1 expression and glucose uptake on macrophages.^57^ Besides, all 12 enzymes necessary for glycolysis (such as LDHA, HK2, and PFK1) are also regulated by HIF-1α.^58^ In animal model, LPS and IFNγ combined treatment also appeared to stabilize the HIF-1α and induce metabolic reprogramming to glycolysis in M1 macrophages.^59^

Interestingly, we found that reduced ERK activity may have initiated decrease of the HIF-1α in M1 macrophages after RBA treatment (Fig. 6). It is known that ERK is part of the RAS/RAF/MEK/ERK kinase cascade.^50,60^ Activated ERK not only increases HIF-1α translation but may also enhance its transcriptional activation.^30^ As ERK knock down showed highly similar effect on M1 macrophages as RBA-NPs treatment, ERK could potentially be a valuable drug target for RA treatment that shares the therapeutic mechanism as RBA. Though there were earlier reports that have identified ERK inhibitors as possible drugs for RA and ERK is known to play role in metabolic reprogramming in cancer, we are the first to find the macrophage polarization related function of ERK in RA.^61,62^ Nevertheless, the metabolic change during/after macrophage polarization is highly complex, and much is still to be investigated. Better understanding of the interactions between energy metabolism and macrophage polarization may help discovering more therapeutic agents for RA that rely on different mechanisms from current medications.

To what extent the tissue damage caused by RA is irreversible, is an interesting question. In this study, it is rather noticeable that RBA-NPs partially repaired the existing bone damage (Fig. 4). In RA patients, secondary osteoporosis is common because the increased number of osteoclasts severely disrupts the osteoclasts-osteoblasts balance and enhances bone resorption. Thus, the M1-to-M2 repolarization of joint macrophages appeared to arrest the ongoing bone resorption and enable the natural repair process of damaged bones. Consistently, M2 macrophages could produce anti-inflammatory cytokines and improve tissue remodeling in RA.^19^ Thus, our study both expands the medical indication of RBA, and supports a way to treat the secondary disease in RA. Nonetheless, how the rebalancing of macrophages affects the number of osteoclasts and osteoblasts remains to be explored.

In conclusion, we constructed a self-assembled nanomicelle with pH-sensitivity and CD44/folate receptor targeting capability to deliver RBA. The designed micelles with hydrophobic cores efficiently encapsulated RBA. RBA-NPs displayed significantly enhanced accumulation in arthritis joints and strong overlapping with M1 macrophages. As a result, RBA-NPs offered strong anti-RA therapeutic outcome by reprogramming M1 to M2 macrophages to restrain the expression of inflammation cytokines and promote tissue repair. We also discovered that RBA-NPs induced this M1-to-M2 phenotypic switch via blocking ERK/HIF-1α/GLUT1 pathway. Therefore, in this study, we fabricated an effective anti-RA nano therapeutic agent based on RBA, revealed its mechanism, and provided an example to reversely modify cell type by regulating metabolic pathways.

## Methods

### Materials

Roburic acid (RBA, CAS: 6812-81-3; catalog B20723) was obtained from Shanghai yuanye Bio-Technology Co., Ltd (Shanghai, China). Sodium hyaluronic acid (HA, molecular weight: 11.5 Kd) was purchased from Freda Biopharm Co. Ltd (Shandong, China). Folic acid (FA) was purchased from Aldrich Chemical Co. (St. Louis, MO, USA). NH_2_-PEG-FA and Fmoc-PEG-OH was purchased from Ruixi Biotech Co. Ltd.

### Animals and cells

Healthy male Sprague-Dawley rats (180–220 g) and male BALB/c mice (18–22 g, 6 weeks old) were purchased from Chengdu Dashuo Experimental Animal Co., Ltd. (Chengdu, China). Murine were kept in a room with controlled temperature (24 ± 2 °C) and humidity (55%) with free access to food and water. The animal experiments in this study were conducted in line with China’s national statute regarding the experimental animals and were approved by Sichuan University’s Institutional Animal Care and Ethics Committee. The murine RAW264.7 cell line was purchased from American Type Culture Collection (ATCC, USA). Dulbecco’s modified Eagle Medium (DMEM) with 1% penicillin and streptomycin was used as cell culture medium, which included 10% fetal bovine serum (GIBCO, USA). The cells were incubated at a 37 °C incubator with 5% CO_2_.

### Synthesis of FA-HA-PAE polymer

The synthesis of FA-HA-PAE polymer involved three steps.

Step 1: HA (500 mg), NH_2_-PEG-FA (2.5 eq.), EDC (4.0 eq.) and NHS (4.0 eq.) were dissolved in 40 °C anhydrous formamide (15 mL) and reacted overnight at 40 °C. The reaction solution was poured into a large amount of acetone to precipitate, and the FA-HA was obtained by filtering.

Step 2: Fmoc-PEG-OH (2 g), acryloyl chloride (2.0 eq.) and triethylamine (2.0 eq.) were dissolved in 20 mL of chloroform and stirred at room temperature for 12 h, followed by washing three times in water. The products were dried with anhydrous sodium sulfate. After decompression concentration, a large amount of ice ether was poured into precipitation, and the products were collected by centrifugation. The Fmoc-PEG-propylene were obtained by vacuum drying. Poly (β-amino ester) (PAE) was synthesized via Michael-addition polymerization. Fmoc-PEG-propylene (1 g), 1,6-bis (acryloyloxy) hexane (10.0 eq.) and 1,3-bis-(4-piperidine) propane (11.0 eq.) were dissolved in 20 mL of chloroform and stirred at 55 °C for 48 h. After decompression and concentration, the reaction solution was poured into a large amount of ice ether to precipitate, and the products were filtered and collected. The Fmoc-PEG-PAE were obtained by vacuum drying. Fmoc-PEG-PAE (1 g) and piperidine (3 mL) were dissolved in 10 mL chloroform and stirred at room temperature for 1 h, followed by washing three times in water. The product was dried with anhydrous sodium sulfate. After decompression concentration, a large amount of ice ether was poured into precipitation, and the products were filtered and collected. The NH_2_-PEG-PAE products were obtained by vacuum drying.

Step 3: FA-HA (500 mg), NH_2_-PEG-PAE (6.0 eq.), EDC (7.5 eq.) and DMAP (0.5 eq.) were dissolved in 40 °C anhydrous formamide (15 mL) and reacted overnight at 40 °C. The reaction solution was poured into a large amount of acetone to precipitate. The FA-HA-PAE was obtained by filtering.

### Preparation and characterization of FA-HA-PAE nanoparticles (NPs) and RBA-loaded FA-HA-PAE nanoparticles (RBA-NPs)

The micelles could be fabricated through the self-assembly ability of FA-HA-PAE polymers. 5 mg RBA and 15 mg FA-HA-PAE copolymer (RBA:FA-HA-PAE copolymer = 1:3, w-w) were respectively dissolved in 5 mL of DMSO and 30 mL of deionized water and then mixed. The mixture was emulsified by a probe-type ultrasonicator (Scientz, Ningbo, China) at 200 W for 10 min to obtain RBA-NPs. To remove the unloaded drugs and excess DMSO solvent, RBA-NPs were loaded into a dialysis bag (MWCO = 7000 Da) against deionized water for 24 h. The blank NPs were prepared in the same method, except adding RBA. The particle sizes and zeta potentials of blank NPs and RBA-NPs were characterized by dynamic light scattering (DLS) (Malvern ZetaSizer Nano ZS90, UK), and transmission electron microscopy (TEM) (H-600, Hitachi, Japan) was used to monitor their morphologies. The concentration of RBA was determined by UV-vis spectrophotometer (Lambda 365, PerkinElmer, USA) with full wavelength scanning, with the maximum absorption wavelength at 210 nm. In the case of DiD-loaded NPs, the lipophilic dye DiD was used in place of RBA.

### The cumulative release of RBA

The cumulative release of RBA was measured using a dynamic dialysis technique in different pH conditions. RBA-NPs (1 mg) were placed into a dialysis bag (MWCO = 7000 Da) and then submerged into 20 mL of PBS buffer within 0.5% Tween 80 at pH 7.4, 6.8 and 5.0 in turns. At certain time intervals (0.5, 1, 2, 4, 6, 12, 24, 36, 48 and 72 h), 1 mL of the release media was withdrawn and replaced with an equivalent volume of fresh media. The concentrations of released RBA were then determined by UV-vis spectrophotometer.

### Cellular uptake study

RAW264.7 cells were seeded in 12-well plates at a density of 1×10^6^ cells per well with or without LPS (100 ng/mL) and IFN-γ (20 ng/mL) and cultured at 37 °C for 24 h. The cell culture media were changed with 1 mL of fresh media containing DiD-NPs (1 μg/mL DiD). After 2 h incubation, cells were washed 3 times by PBS. A flow cytometer (BD FACSCelesta, USA) was used to quantitatively analyze the fluorescence intensity of DiD. Cells were fixed and stained with DAPI, and then photographed by laser scanning confocal microscope (LSM 800, Zeiss, Germany).

### AIA model

The rats’ base of tails were injected subcutaneously with Complete Freund’s adjuvant (80 μL) containing 10 mg/mL heat-killed mycobacteria (Chondrex, #7027, Washington DC, USA). The development of arthritis progression was tracked on a daily basis and was fully established at 14 days after injection.

### Immunofluorescence staining

Tail veins of AIA rats were administered with free DiD or DiD-NPs. Ankle joints were collected 24 h after the last administration to prepare sections. The prepared sections of 10 μm thick slices were stained with CD44 antibody (Affinity Biosciences, DF6392, 1:500), CD68 antibody (Affinity Biosciences, DF7518, 1:500), and FOLR2 antibody (Affinity Biosciences, DF9518, 1:300). Nuclei was stained by DAPI. A laser scanning confocal microscope (LSM 800, Zeiss, Germany) was taken to record the fluorescent distributions in synovial joints.

### Biodistribution in AIA rats

Tail veins of AIA rats were injected with free DiD or DiD-NPs. After administration, the biodistribution of DiD in the ankle joints was tested by in vivo imaging measurement of fluorescence intensity using a Caliper IVIS Lumina III In Vivo Imaging System (Perkin Elmer, USA) at specific time points (0.5, 2, 6, 12, 24, and 48 h). After photographing, rats were sacrificed, and hearts, livers, spleens, lungs, kidneys, and paws were collected for in vivo imaging. Image J (National Institutes of Health, USA) was used to quantify the corresponding fluorescence intensity. AIA rats treated with N.S. were set as controls.

### Polarization of M1 and M2 phenotype macrophages

M1 macrophage polarization was achieved by treating RAW264.7 with LPS (100 ng/mL) and IFN-γ (20 ng/mL) for 24 h. M2 macrophage polarization was achieved by treating the RAW264.7 with IL-4 (20 ng/mL) + IL-13 (20 ng/mL) for 24 h. THP-1 cells were induced to differentiate into macrophages by adding phorbol myristate acetate (PMA) 100 ng/ml for 48 ~ 72 h. M1 type macrophages were used LPS (100 ng/mL)+IFN- γ (20 ng/mL) treatment for 48 h; M2 type macrophages were used IL-4 (20 ng/mL)+IL-13 (20 ng/mL) for 48 h. THP-1 cells are adherent cells after differentiation. The shift of M1-to-M2 macrophages was induced by RBA-NPs (the equivalent of 20 μM of RBA) for 24 h. Immunofluorescence staining and flow cytometry were used to reveal the frequencies of M1 and M2 macrophages. The levels of M1(iNOS, TNF-α, IL-1β) and M2 (Arg-1, IL-10, TGF-β) markers were determined utilizing ELISA assay. The cells were blocked by blocking buffer (PBS solution with 5% BSA) and fixed by 4% paraformaldehyde, and then incubated with primary antibodies against PE anti-mouse CD68 antibody (Biolegend, 137013, 1:200), PE anti-mouse F4/80 Antibody (Biolegend, 123109, 1:100), PE anti-human CD68 Antibody (Biolegend, 333807, 1:20), FITC anti-mouse CD86 antibody (Biolegend, 105005, 1:100), APC anti-mouse CD206 (MMR) antibody (Biolegend, 141707, 1:100), FITC anti-human CD86 Antibody (Biolegend, 374203, 1:20) and APC anti-human CD206 (MMR) antibody (Biolegend, 321109, 1:50) at 4 °C overnight. After being counterstained with DAPI, the laser scanning confocal microscope (LSM 800, Zeiss, Germany) was used to image the sections.

### Flow cytometry

For intracellular staining, the cells were blocked by blocking buffer (PBS solution with 5% BSA) and incubated with FITC anti-mouse CD86 antibody (Biolegend, 105005, 1:100) and APC anti-mouse CD206 (MMR) antibody (Biolegend, 141707, 1:100) at 4 °C for 1 h in a dark place. The cells were then washed in PBS and detected by a flow cytometry (BD FACSCelesta, USA). The results were analyzed using FlowJo software (FlowJo LLC, Ashland, OR, USA).

### Therapeutic efficacy evaluation

The forty rats were divided into six groups at random (*n* = 8): Normal, AIA rats with N.S. (N.S.), AIA rats with dexamethasone (0.5 mg/kg) (Dex), AIA rats with free RBA (5 mg/kg) (RBA), AIA rats with blank NPs (Blank NPs), or AIA rats with RBA-NPs (dose of 5 mg/kg for RBA) (RBA-NPs) with intravenous administration. After arthritis induction, treatment was given on days 17, 20, 23, and 26. In the N.S. group, AIA rats were given with an equal volume of saline. During treatment, the paw thickness of ankle joints was assessed every two days. On day 14, each hind limb was graded on a 0 to 4 scale: 0 means normal; 1 means slight erythema and/or swelling; 2 means moderate redness and swelling; 3 means severe swelling; 4 means ankylosis and inability to bear weight. The limb scores of each mouse were added together to yield a maximal score of 16. On day 28, the rats were sacrificed via anesthesia (pentobarbital sodium, 65 mg/kg, intraperitoneal). The thymus and spleen were taken and weighed right away. The thymus and spleen indices were calculated respectively by dividing the wet weight of the thymus and spleen by body weight (mg/10 g).

### Histopathological examination

Twenty-eight days after arthritis induction, all of the rats were sacrificed. The ankle joints were removed and fixed with 4% paraformaldehyde. Ankle joints were decalcified and fixed using 15% tetrasodium ethylenediaminetetraacetic acid for 2 months. Sections were cut at 3 µm thicknesses after processing for paraffin embedding, followed by stained with hematoxylin-eosin (HE) and Safranin-O Fast-Green. A light microscope (Olympus BX53, Tokyo, Japan)was used to observe staining.

### Immunohistochemical analysis and TRAP assay

4% paraformaldehyde was applied to fix ankle joints collected before and two days after the last treatment. A 15% tetrasodium ethylenediaminetetraacetic acid solution was then used to decalcify the fixed ankle joints were decalcified with daily changes of a 15% (w/v) tetrasodium ethylenediaminetetraacetic acid solution for 2 months. The decalcified joints were subsequently embedded in paraffin and then sectioned for staining. Commercial streptavidin-biotin complex (SABC) kits (Boster, Wuhan, China) were used to immunolocalize ALP polyclonal antibody (Invitrogen, PA5-106391, 1:200), anti-RANKL (Abcam, ab239607, 1:100), anti-OPG (Abcam, ab203061, 1:200), IL-6 Polyclonal antibody (Proteintech, 23457-1-AP, 1:100), IL-1 beta Polyclonal antibody (Proteintech, 26048-1-AP, 1:100), TNF Alpha Monoclonal antibody(Proteintech, 60291-1-Ig, 1:500) in the joints. The TRAP staining kit (Wako Pure Chemical Industries, Osaka, Japan) was applied to stain these sections following the instructions.

### Micro-CT imaging

All rats were sacrificed on day 28 after arthritis induction. After fixed in paraformaldehyde at a concentration of 4%, an ex vivo micro-computed tomography (Micro-CT, SCANCO MEDICAL VivaCT 80, Switzerland) was used to scan the ankle joints were scanned at 70 kV and 113 μA with a 15 μm resolution. The 3D pictures of the distal femur’s joints and trabecular were created by rebuilding the dataset. Furthermore, quantitative analyses were performed for certain morphometric characteristics such as bone mineral density (BMD), bone surface vs. bone volume (BS/BV), trabecular separation (Tb.Sp), and trabecular bone thickness (Tb.Th).

### Safety assessment

Healthy rats (200 ± 20 g) were intravenously injected with 5 mg/kg of RBA, blank NPs, or RBA-NPs to investigate the in vivo safety of RBA-NPs. Rats treated with an equivalent volume of saline were set as the N.S. group. The rats were slaughtered two days following the final dose, and key organs including the heart, liver, spleen, lung, and kidney were removed for histological analysis as previously reported. The levels of alanine aminotransferase (ALT), aspartate aminotransferase (AST), creatine kinase (CK) and lactic dehydrogenase (LDH), creatinine (CREA), uric acid (UA), white blood cell (WBC), red blood cell (RBC), and platelet (PLT) in the obtained serum from the administered rats were assayed on a Hitachi 7020 automatic biochemical analyzer (Hitachi, Japan). Lung tissue edema was evaluated by the ratio of lung W/D. The lung tissues were divided from the left upper lung lobe. After remove the water, the tissues were weighed firstly and reweighed followed dehydration at 80 °C for 24 h. The results were concluding as the wet weight divided by the dry weight.

### Protein extraction

The RAW264.7 cells were washed three times in pre-cooled PBS before being centrifuged at 1000 g for 5 min at 4 °C. After removing supernatant, samples were incubated for 5 min in Lysis buffer (40 mM Tris-HCl, 4% SDS, 2 M Thiourea,7 M Urea, pH 8.5) containing 2 mM EDTA, 1 mM PMSF, and 10 mM DTT. The suspension was then sonicated on ice for 5–15 min before being centrifuged for 20 min at 13,000 rpm, 4 °C. A pre-cooled acetone solution of four volumes was added to the supernatant at −20 °C for 2 h. Centrifugation of the protein pellets and resuspension in a urea/TEAB solution containing 8 M urea and 100 mM TEAB (pH 8.0) were followed by air drying. Protein samples were reduced for 30 min at 56 °C with 10 mM DTT, then alkylated for 30 min at room temperature in the dark with 50 mM iodoacetamide (IAM). 4 volumes of precooled acetone at −20 °C for 2 h were added to the sample for centrifugation. The air-dried protein pellets were resuspended in the above-mentioned urea/TEAB solution. The concentration of total protein was assessed according to the Bradford method. For tryptic digestion, an equivalent amount of protein from each sample (about 100 μg) were employed. The trypsin was introduced at a 1:50 (w/w) enzyme-to-protein ratio. Following digestion at 37 °C for 12–16 h, C_18_ columns were used to desalt the peptides which were then dried with a vacuum concentration meter. Trypsin was introduced at an enzyme-protein ratio of 1:50 (w/w), and the digestion was carried out at 37 °C for 12–16 h. Peptides were desalted using C_18_ columns after digestion, and the desalted peptides were dried using a rotary evaporator.

### Bioinformatics analysis

Heat maps were drawn using Perseus (1.6.2.2). GeneCodis 3.0 was used for GO and KEGG pathway analysis. FDR (*q*-value) was used to select proteins of interest. In the quantitative results, the changed protein was considered to be differentially expressed when the fold change of the protein was >1.5 or <0.667 and the *p*-value was <0.05.

### Statistical analysis

The quantitative results were provided as mean ± standard deviation. A Student’s two-sided *t*-test was used for statistical analysis of a two-group comparison. For multiple comparisons, a two-way analysis of variance (ANOVA) was utilized. A significant difference was considered at *P*-value < 0.05.

## Supplementary information

Metabolic reprogramming of proinflammatory macrophages by target delivered roburic acid effectively ameliorates rheumatoid arthritis

Supplementary materials 1

Supplementary materials 2

## Data Availability

The authors declare that the data supporting the findings of this study are available within the paper and its Supplementary materials. All data generated during this study are included in this published article and its supplementary information files. All data in this study are available from the corresponding author with a reasonable request. The mass spectrometry proteomics data have been deposited to the ProteomeXchange Consortium (http://proteomecentral.proteomexchange.org) via the iProX partner repository^63,64^ with the dataset identifier PXD042274.
