# Supplementary material for: Metabolic reprogramming of proinflammatory macrophages by target delivered roburic acid effectively ameliorates rheumatoid arthritis symptoms
Source: Signal Transduct Target Ther. 2023 Jul 28;8:280. doi: 10.1038/s41392-023-01499-0 (PMC10374631; doi:10.1038/s41392-023-01499-0)
Supplement: Supplementary file 1 — Metabolic reprogramming of proinflammatory macrophages by target delivered roburic acid effectively ameliorates rheumatoid arthritis [file 41392_2023_1499_MOESM1_ESM.docx]

Supplementary Materials for

Metabolic reprogramming of proinflammatory macrophages by target delivered roburic acid effectively ameliorates rheumatoid arthritis

Na Jia ^a^, Yunzhen Gao ^a^, Min Li ^a^, Yi Liang ^a^, Yuwen Li ^b^, Yunzhu Lin ^a^, Shiqi Huang ^a^, Qing Lin ^a^, Xun Sun ^a^, Qin He ^a^, Yuqin Yao ^a^, Ben Zhang ^a^, Zhirong Zhang ^a^, Ling Zhang ^a,^*

Correspondence to: zhangling83@scu.edu.cn

**This PDF file includes:**

Materials and Methods

Figures. S1 to S31

Tables S1 to S5

Materials and Methods

1. Establishment of FA-HA-PAE *in vitro* analysis methods

1.1 The HPLC analysis

The concentrations of FA-HA-PAE were determined by High Performance Liquid Chromatography (HPLC). The instrument was 1260 Infinity HPLC (Agilent Technologies, USA) with the Agilent HC-C_18_ column. The detection wavelength was adjusted to 229 nm, and the mobile phase was acetonitrile-ultrapure water (95:5). Adjusted the flow rate as 1 mL/min, the intake volume as 1 μL and the column temperature as 25℃. The HPLC chromatogram of FA-HA-PAE were recorded as Supplementary Figure. S5.

1.2 Establishment of the standard curve

100 mg of FA-HA-PAE was fully dissolved with the mixed solution as mobile phase to 100 mL to obtain 1 mg/mL FA-HA-PAE reserve solution. Gradient solutions of 100, 50, 25, 12.5, 6.25 and 3.125 mg/mL were prepared. 1 μL of each concentration was detected by HPLC, and each sample was tested three in parallel. According to the above HPLC analysis conditions, the concentration (C, mg/mL) is the X-axis and the absorbance (A) is the Y-axis. Linear regression is performed to establish FA-HA-PAE standard curve. The results were shown in Figure. S6, and the standard curve equation is: A=22.699C+5.0928, R^2^=0.9999. The results showed a good linear relationship between FA-HA-PAE concentrations within the range of 3.125-100 mg/mL.

1.3 Precision

The FA-HA-PAE stock solution was taken using a pipette and prepared into three different concentrations of 3.125, 50 and 100 mg/mL. The absorbance of the three FA-HA-PAE solutions was measured five times in the same day, and the standard curve equation to calculate the FA-HA-PAE concentration. The results are shown in Table S1, the precision of FA-HA-PAE solutions at three concentrations of 3.125, 50 and 100 mg/mL was 0.86%, 2.11% and 1.73%.

1.4 Recovery rate

The FA-HA-PAE stock solution was taken using a pipette and prepared into three different concentrations of 3.125, 50 and 100 mg/mL. The absorbance of the three FA-HA-PAE solutions was measured and taken into the standard curve equation to calculate the FA-HA-PAE concentration. The recovery rate was calculated as the measured concentration/the prepared concentration. The results are shown in Table S3, the average recovery of FA-HA-PAE solutions at three concentrations of 3.125, 50 and 100 mg/mL were 97.30%, 102.06% and 96.19% (n=5), respectively. The corresponding RSD values were 0.95%, 2.12% and 1.42%, respectively. The sample recovery rate determined by this method was in the range of 95%-105%, indicating a compliance with the assay requirements.

1.5 Content detection

We accurately weighed 53.91 mg of FA-HA-PAE and tested the content using HPLC method above. The peak area of FA-HA-PAE was 1195.705, and the content was calculated as 52.45 mg according to the standard curve. The purity of FA-HA-PAE was 97.29% which can be used for subsequent experiments.

2. Establishment of RBA *in vitro* analysis methods

2.1 The UV analysis

The concentrations of RBA were determined by UV-vis spectrophotometer. The UV spectra were recorded between 190 and 800 nm for peak characterization, and the detection wavelength was set at 210 nm. Supplementary Figure. S5 showed the UV absorption spectrum of RBA in acetonitrile with the maximum absorption peak position at 210 nm.

2.2 Establishment of the standard curve

9 mg of RBA was fully dissolved with acetonitrile to 100 mL to obtain 90 μg/mL RBA reserve solution. Gradient solutions of 90, 45, 18, 14.4, 7.2, 3.6, 1.8, and 0.9 μg/mL were prepared. 2 mL of each concentration was placed in a cuvette for absorbance at 210 nm, and each sample was tested three in parallel. According to the above UV analysis conditions, the concentration (C, μg/mL) is the X-axis and the absorbance (A) is the Y-axis. Linear regression is performed to establish RBA standard curve. The results were shown in Supplementary Figure.S6, and the standard curve equation is: A = 0.0069C + 0.0188, R^2^=0.9992. The results showed a good linear relationship between RBA concentrations within the range of 0.9-90 μg/mL.

2.3 Precision

The RBA stock solution was taken using a pipette and prepared into three different concentrations of 45,14.4 and 3.6 μg/mL. The absorbance of the three RBA solutions was measured five times in the same day, and the standard curve equation to calculate the RBA concentration. The absorbance of the three RBA solutions was measured for five consecutive days, and the standard curve equation to calculate the RBA concentration to obtain the daytime precision. The results are shown in Table S2.

2.4 Recovery rate

The RBA stock solution was taken using a pipette and prepared into three different concentrations of 3.6,14.4 and 45 μg/mL. The absorbance of the three RBA solutions was measured and taken into the standard curve equation to calculate the RBA concentration. The results are shown in Table S4, the recovery of RBA solutions at three concentrations of 3.6,14.4 and 45 μg/mL is 100.93%, 99.91%, 100.34%, respectively. The corresponding RSD values are 1.75%, 0.36%, 2.23%, respectively. The sample recovery rate determined by this method was in the range of 95% -105%, and the RSD value was less than 3%, indicating a compliance with the assay requirements.

3. Encapsulation efficiency (EE) and drug loading (DL) for RBA

Encapsulation efficiency (EE) and drug loading (DL) are important indicators to evaluate the drug loading capacity of carrier. The effective separation of NPs and uncoated free RBA is the basis for investigating the EE and DL of RBA-NPs. Ultrafiltration method is used in this experiment. RBA (1.1 mg) and FA-HA-PAE (3.3 mg) were prepared for RBA-NPs according to the preparation method. RBA-NPs were placed on the upper layer of the ultrafiltration tube (with a molecular weight of 3000 Da), and centrifuged at 3000 rpm for 30 min to obtain the separated NPs and free RBA. The lower clarification solution was filtrated by 0.22 μm microporous membrane. The content of unloaded RBA was determined according to the detection method. The formula for calculating the EE and DL of RBA-NMs is as follows:

EE% =$\frac{total mass of RBA-mass of free RBA in the lower solution}{total mass of RBA}\times100$

DL% =$\frac{total mass of RBA-mass of free RBA in the lower solution}{mass of NPs}\times100$

Three samples were tested in parallel. The EE and DL of RBA-NPs was 84.2 ± 1.3% and 9.6 ± 0.8%, respectively.

Figure. S1.


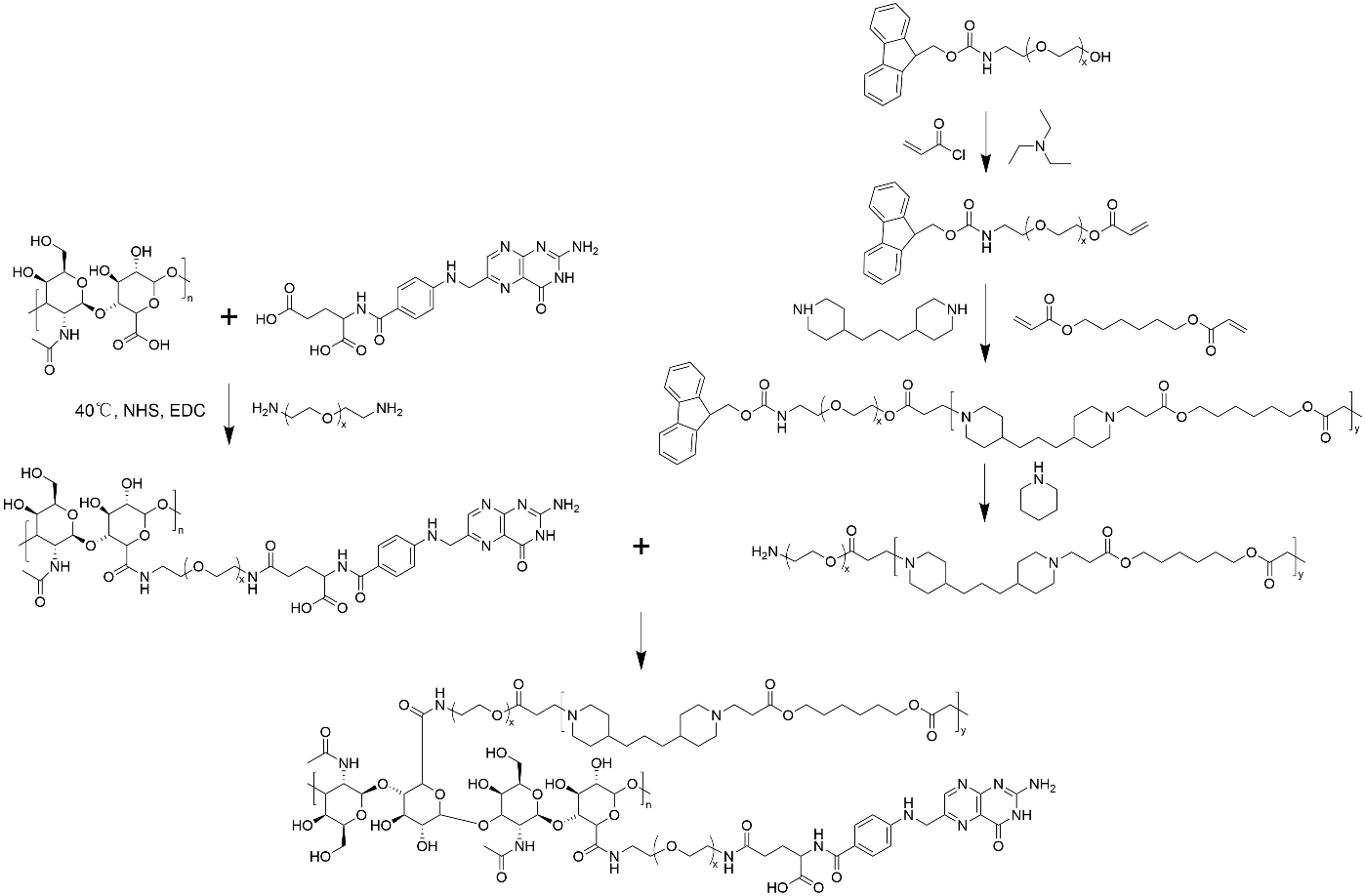


**Figure. S1.** Synthetic scheme of PAE-HA-FA copolymer.

Figure. S2.


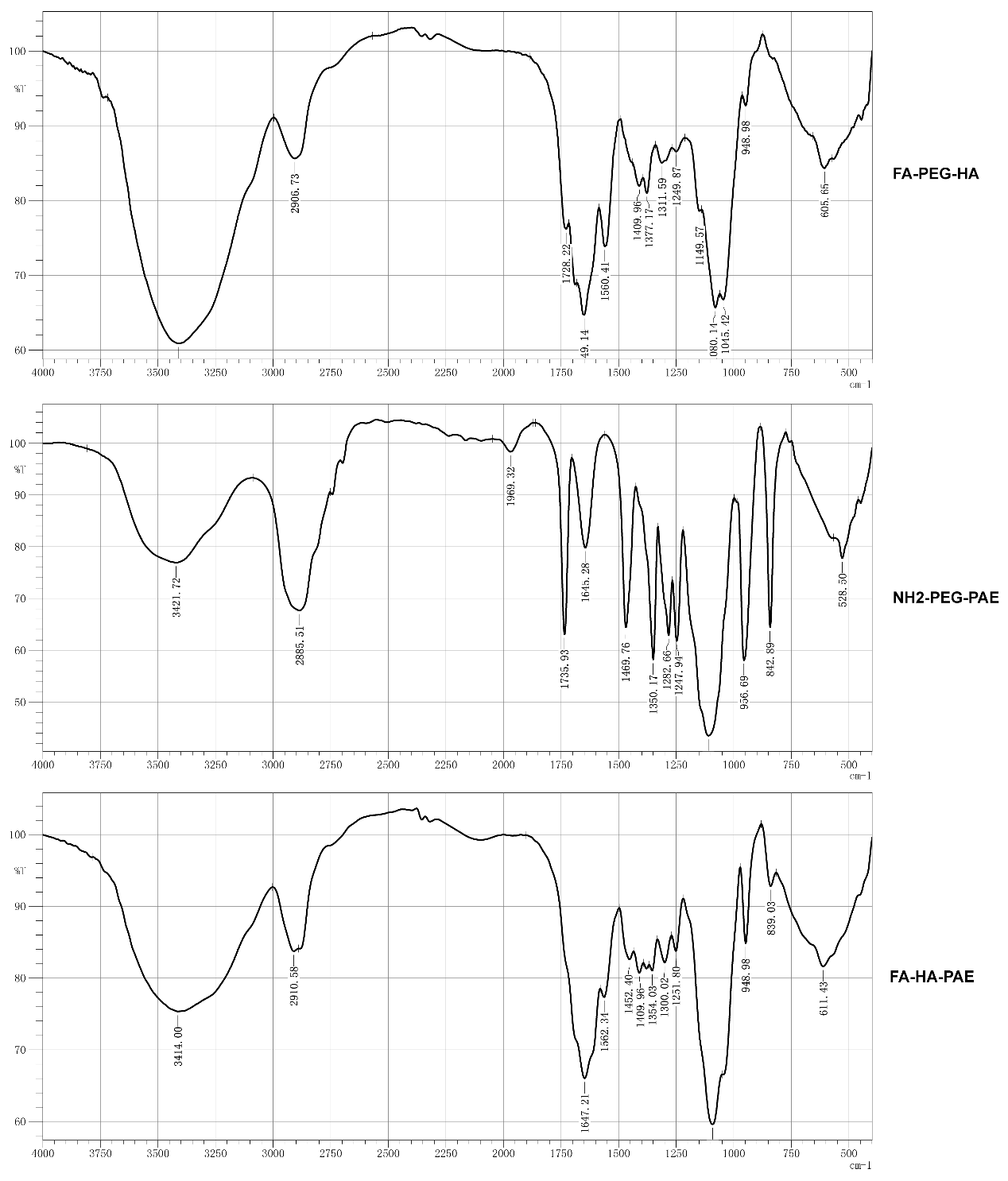


**Figure. S2.** FTIR spectra analysis of PAE-HA-FA graft copolymer.

Figure. S3.


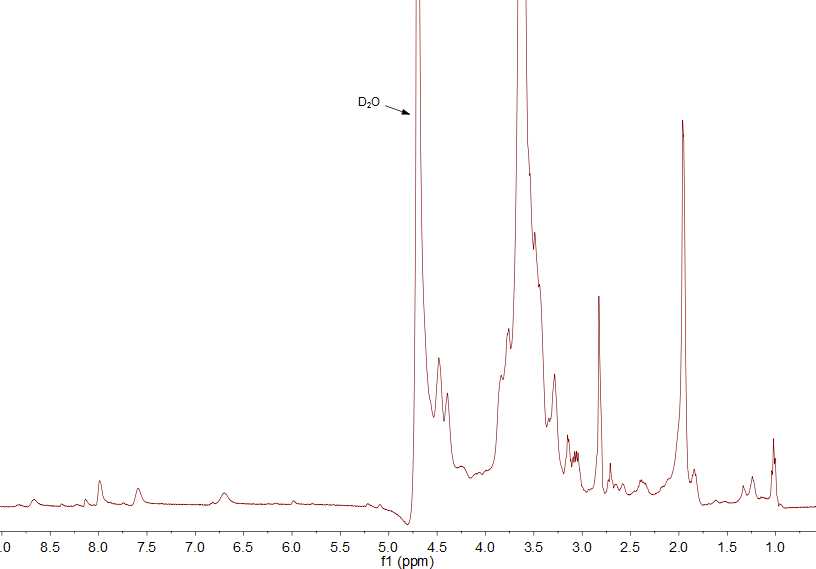


**a**


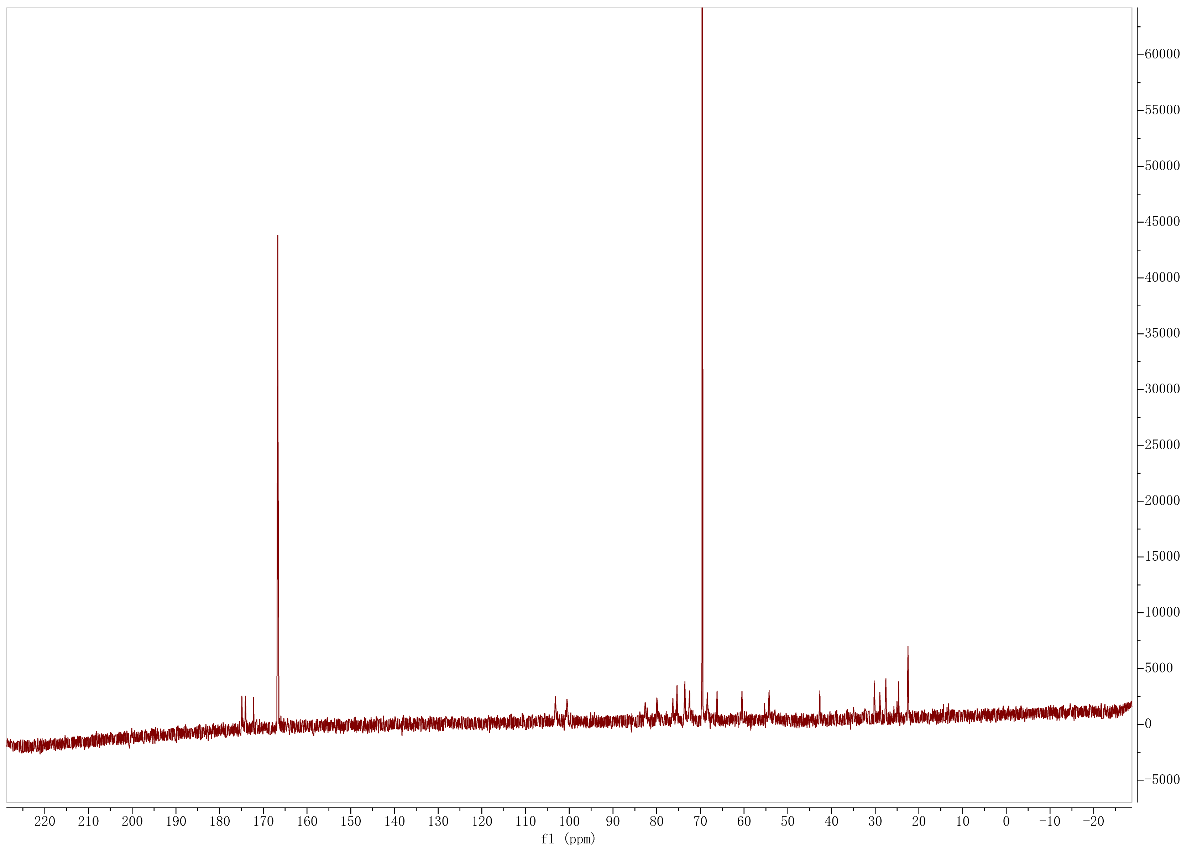


**b**

**Figure. S3.** ^1^H-NMR spectra (a) and ^13^C-NMR (b) spectra of PAE-HA-FA graft copolymer.

Figure. S4.


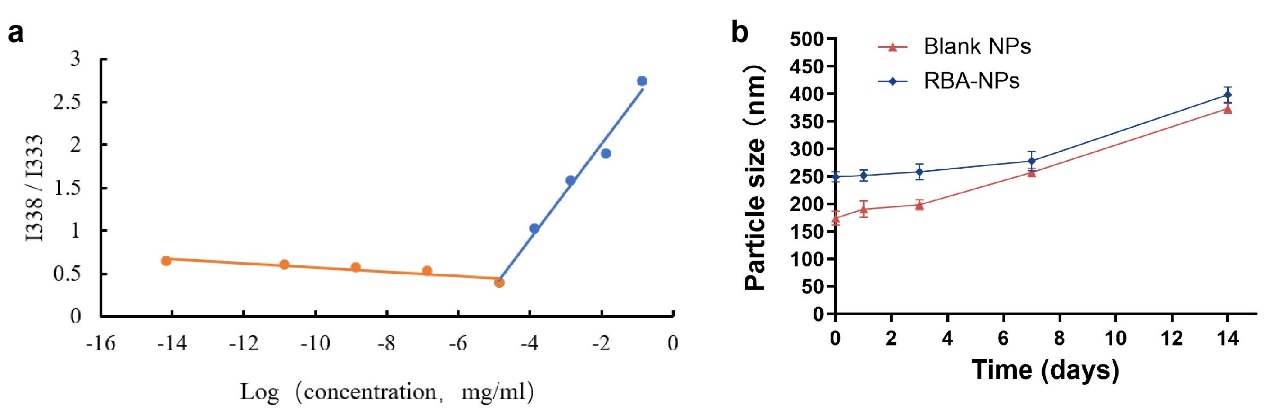


**Figure. S4.** The critical micelle concentration (CMC, a) and serum stability (b) of blank NPs and RBA-NPs.

Figure. S5.


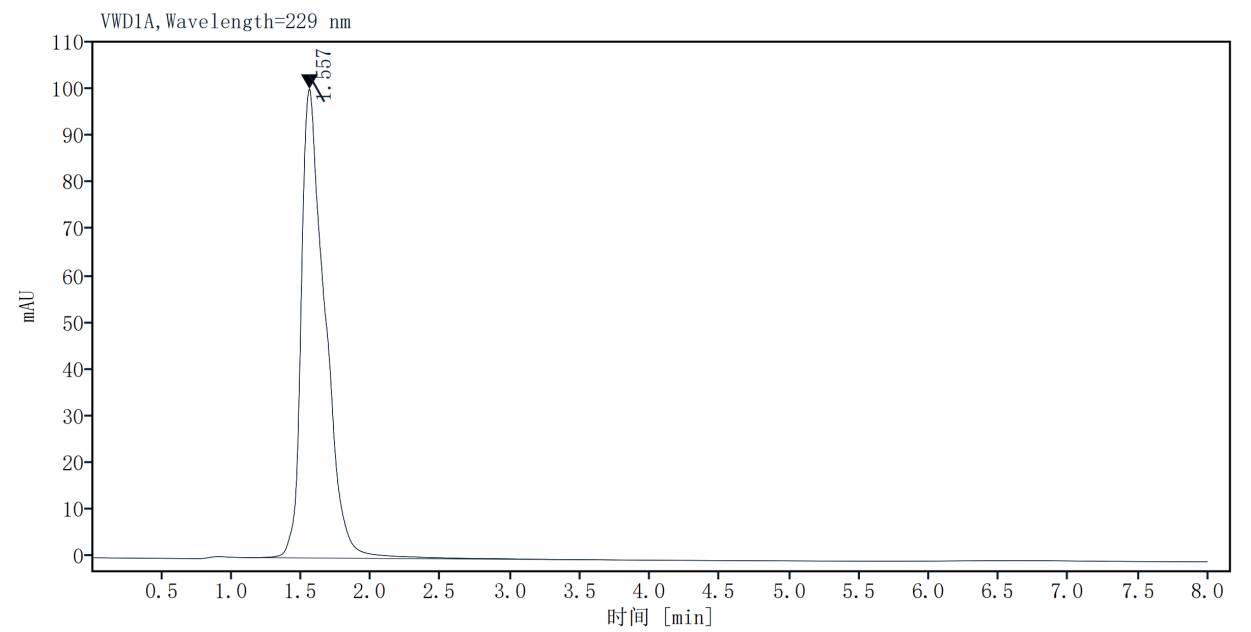


**a**


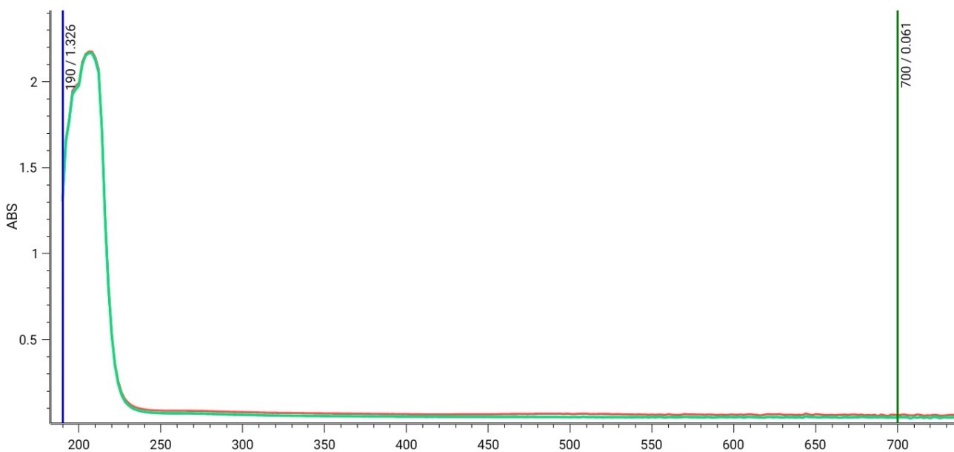


**b**

**Figure. S5.** The HPLC chromatogram of FA-HA-PAE (a) and IV spectra of RBA (b).

Figure. S6.

**a**


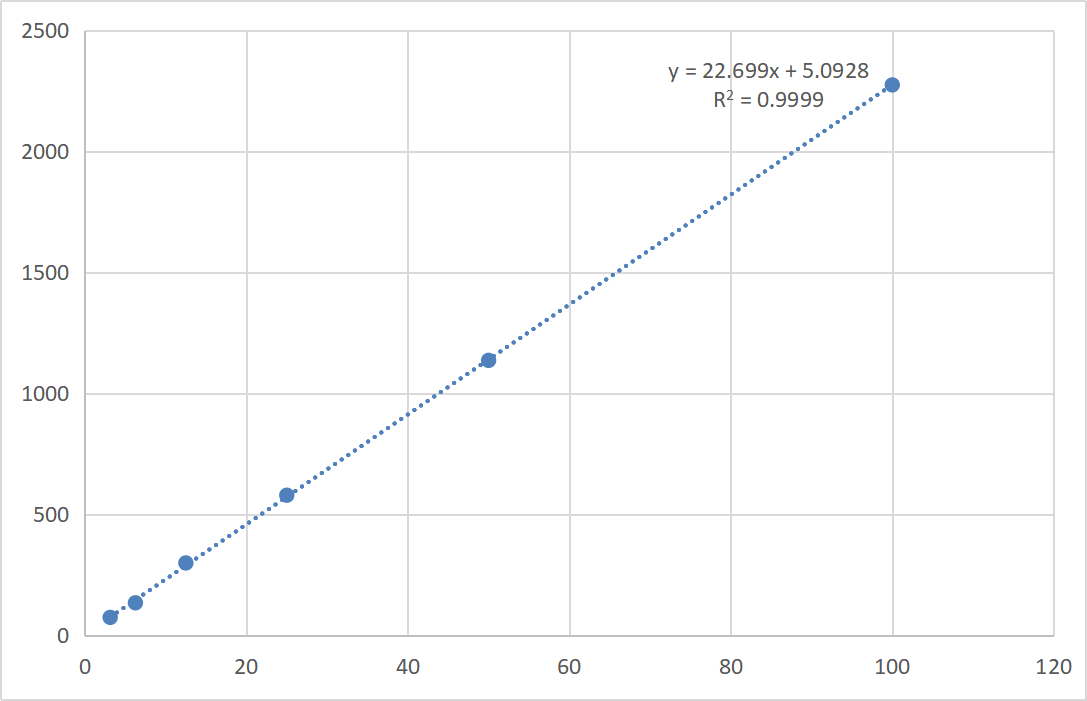


**b**


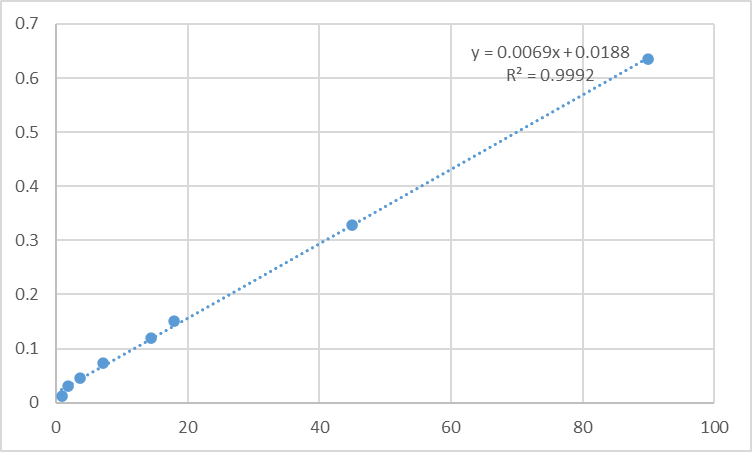


**Figure. S6.** The standard curve of FA-HA-PAE (a) and RBA (b).

Figure. S7.


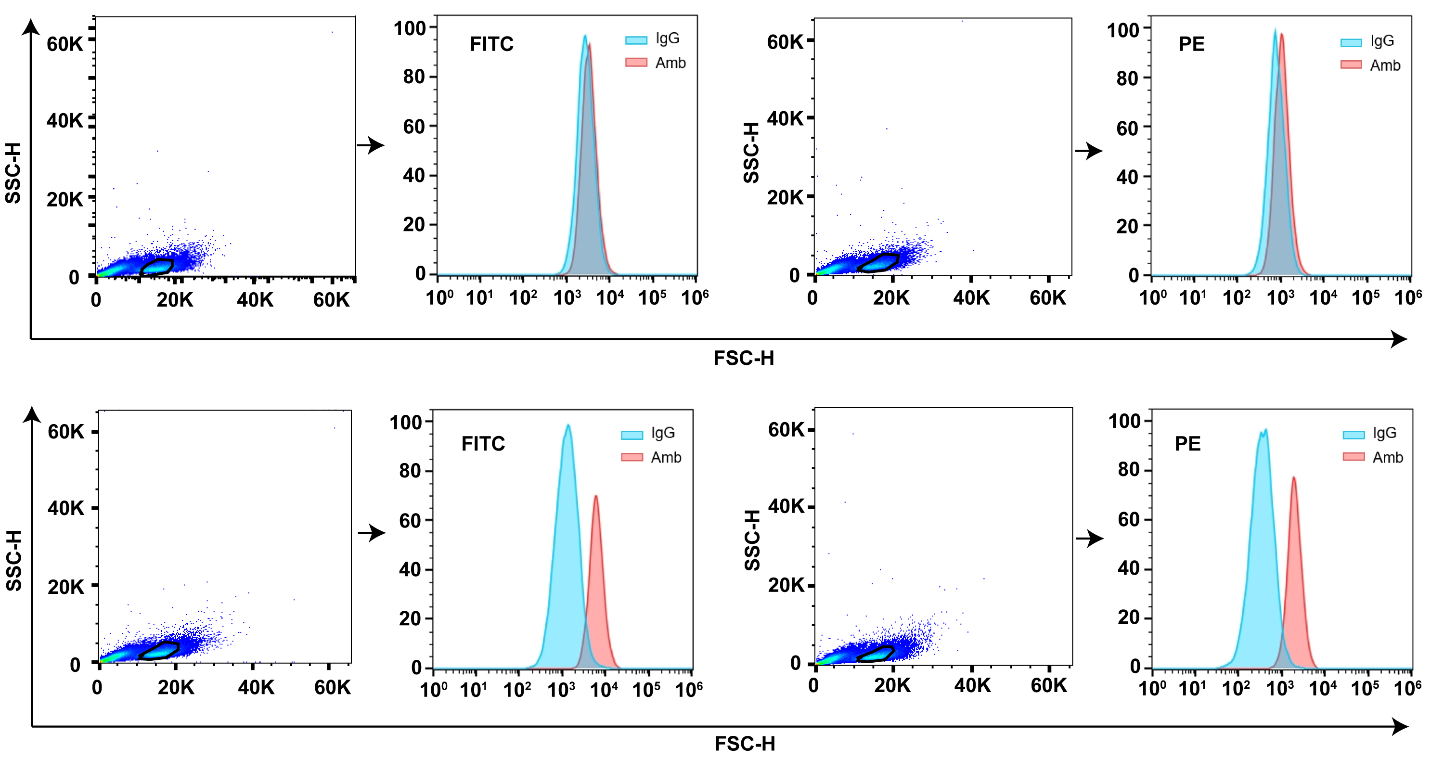


**Figure. S7.** The gating strategies of Figure 2c.

Figure. S8.


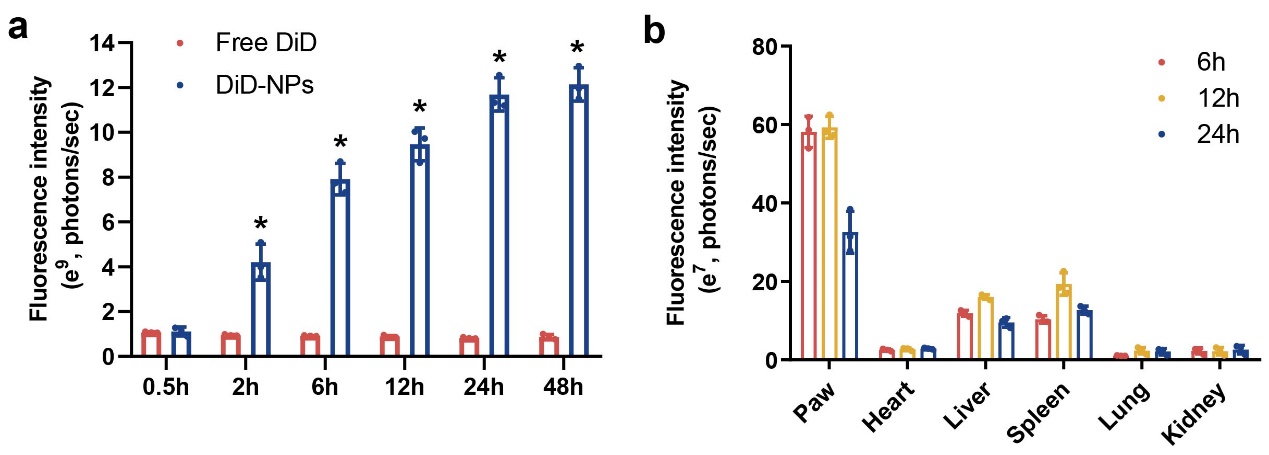


**Figure. S8.** (a) The statistical graphs of the inflamed joint distribution based on the semi-quantitative analysis of the in vivo fluorescence images after i.v. administration of free DiD and DiD-NPs in AIA rats at 0.5, 2, 6, 12, 24 and 48 h. (b) The statistical graphs of the fluorescence intensity of organs based on the semi-quantitative analysis of the ex vivo fluorescence images after i.v. administration of DiD-NPs. Data represent mean ± SD. Statistical significance was determined by two-sided Student’s t-test.Type or paste caption here.

Figure. S9.


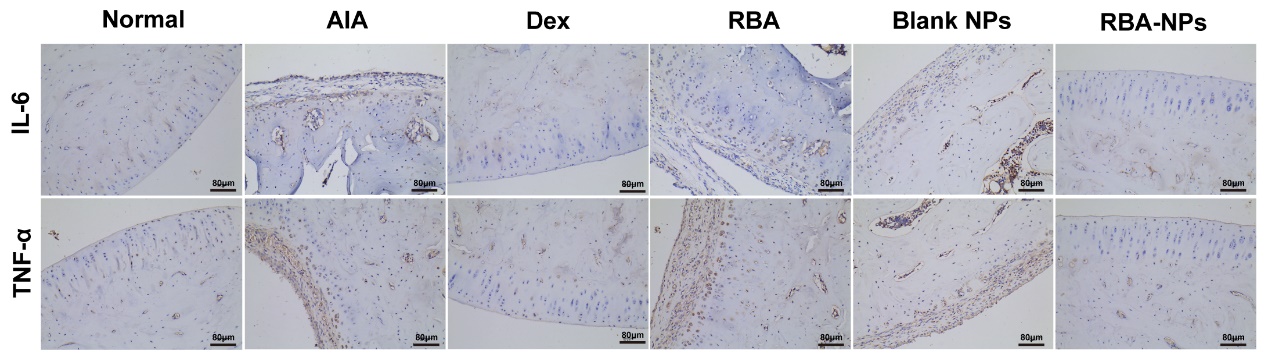


**Figure. S9.** The expression levels of IL-6 and TNF-α in arthritic joints in different groups (Scale bar = 80 μm) (n = 5 independent animals).

Figure. S10.


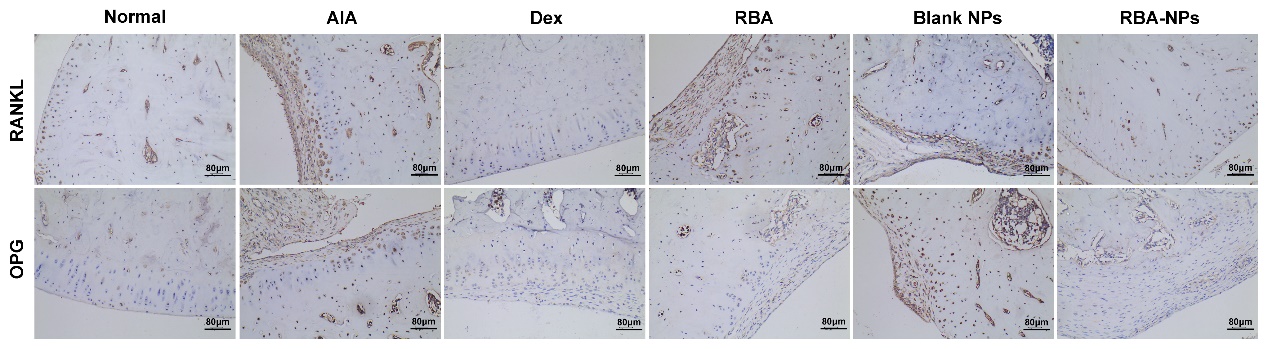


**Figure. S10.** Immunohistochemical analyses of the RANKL and OPG in the joint tissues from rats receiving the indicated treatment (Scale bar =80 μm) (n = 5 independent animals).

Figure. S11.


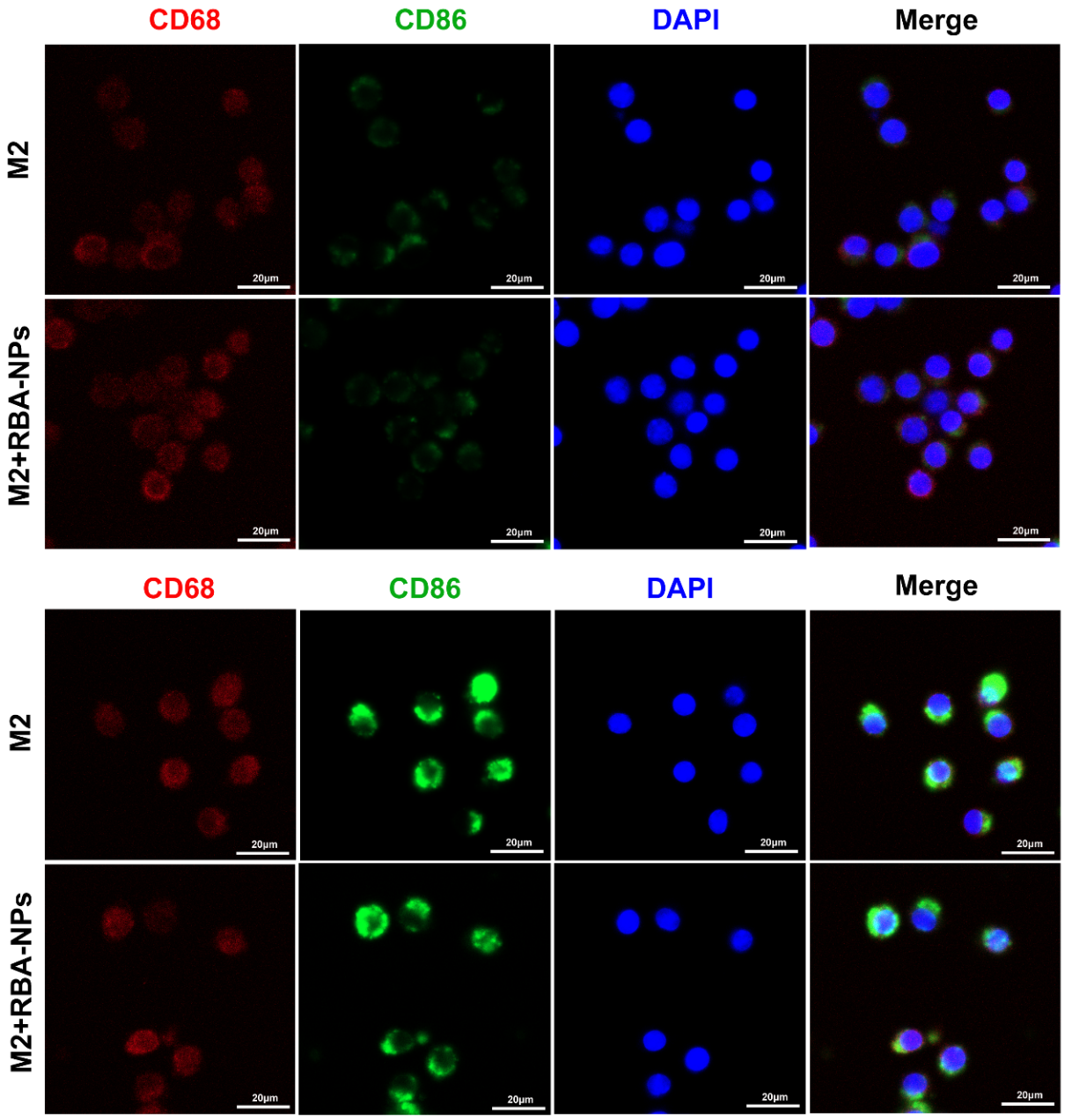


**Figure. S11.** RBA-NPs did not appear to affect the proportion of M2 phenotype macrophages in after IL-4+IL-13 induced RAW264.7 cells. Immunofluorescence staining of CD68 (red), CD86 and CD206 (green), and nuclei (blue) without or with treatment RBA-NPs. Scale bar = 20 μm.

Figure. S12.


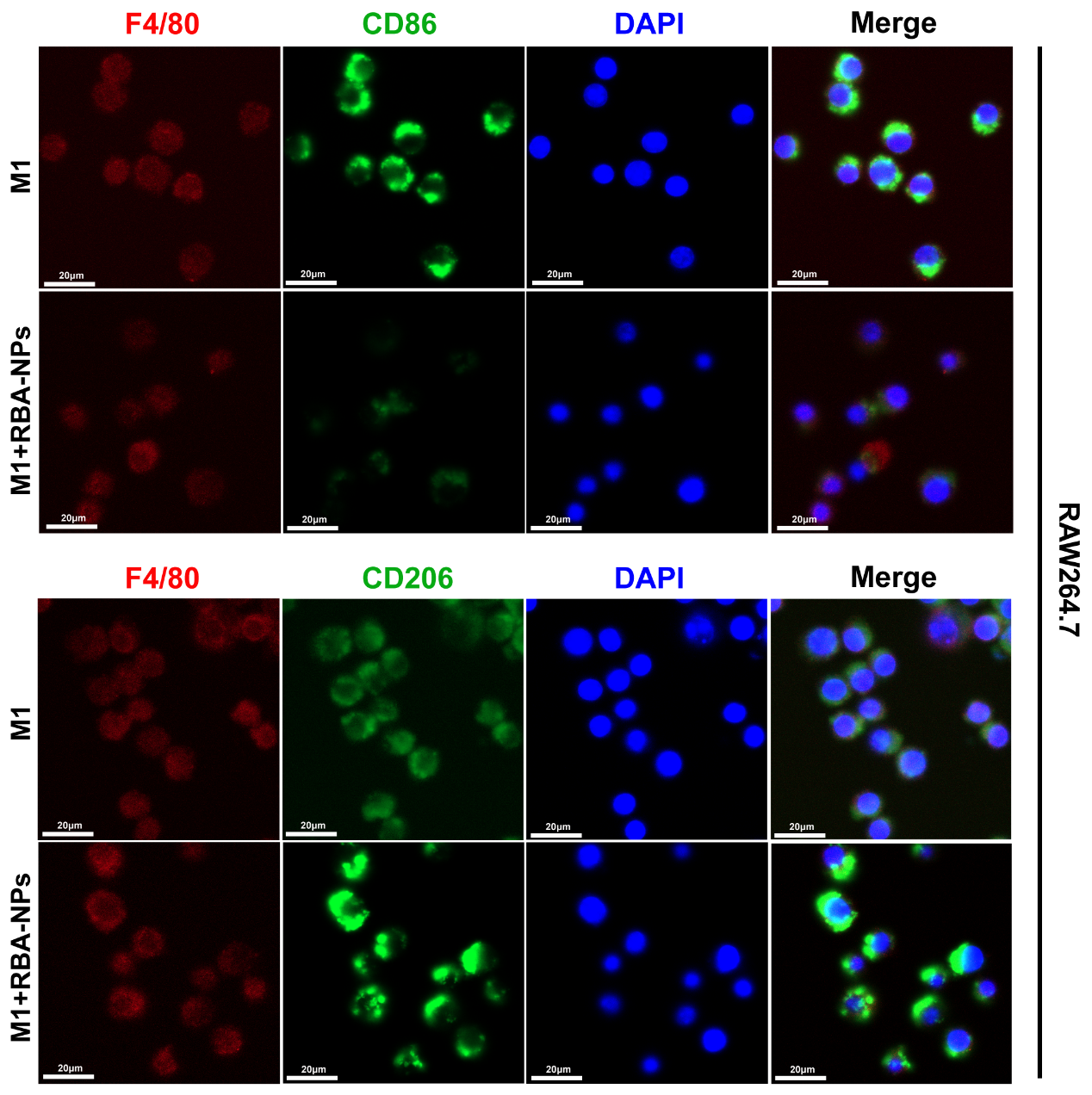


**Figure. S12.** RBA-NPs reprogram proinflammatory M1 macrophages to anti-inflammatory M2 macrophages in RAW264.7 cells. Immunofluorescence staining of F4/80 (red), CD86 and CD206 (green), and nuclei (blue) on LPS+IFN-γ activated macrophages without or with treatment RBA-NPs. Scale bar = 20 μm.

Figure. S13.


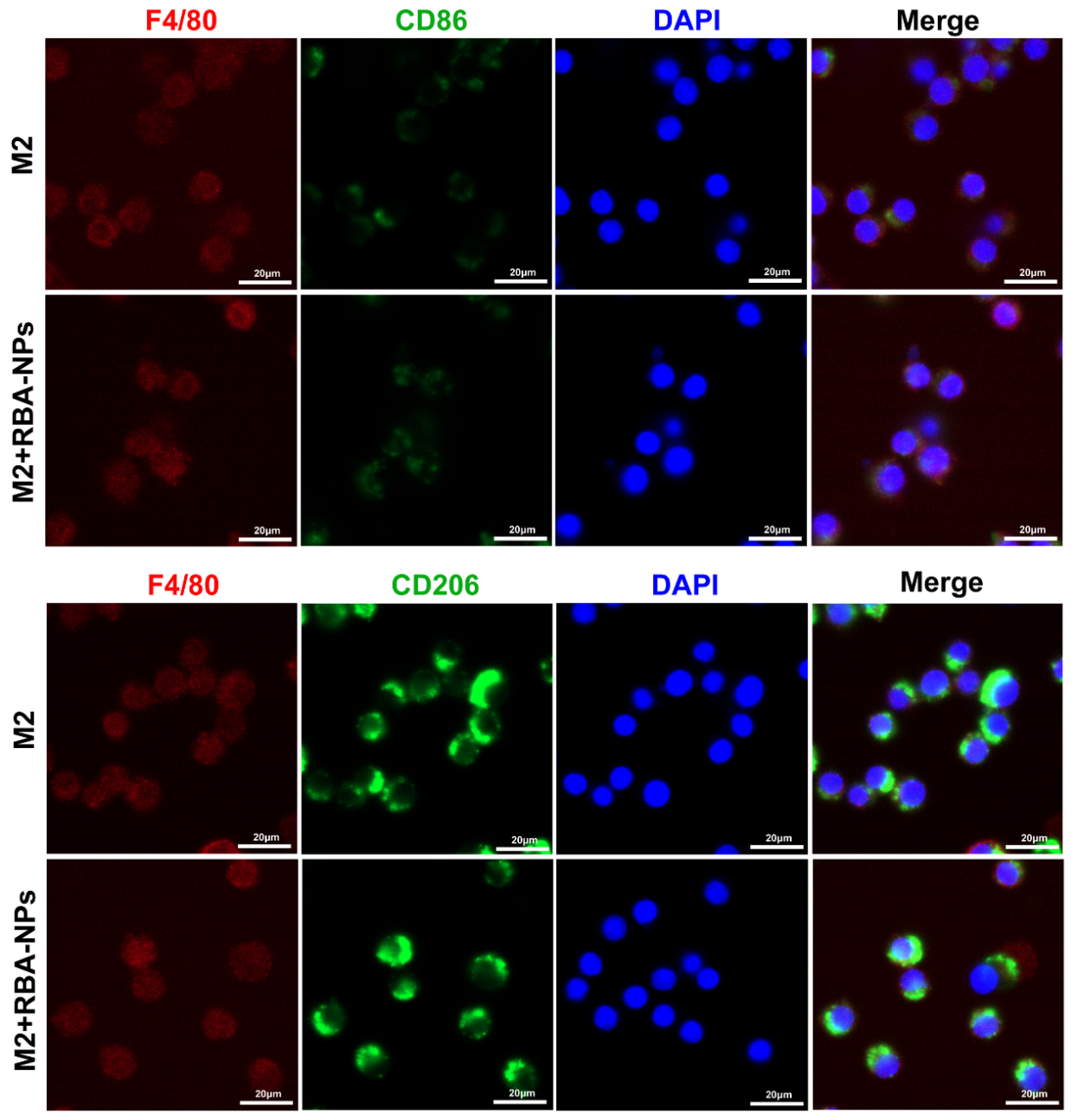


**Figure. S13.** RBA-NPs did not appear to affect the proportion of M2 phenotype macrophages in after IL-4+IL-13 induced RAW264.7 cells. Immunofluorescence staining of F4/80 (red), CD86 and CD206 (green), and nuclei (blue) without or with treatment RBA-NPs. Scale bar = 20 μm.

Figure. S14.


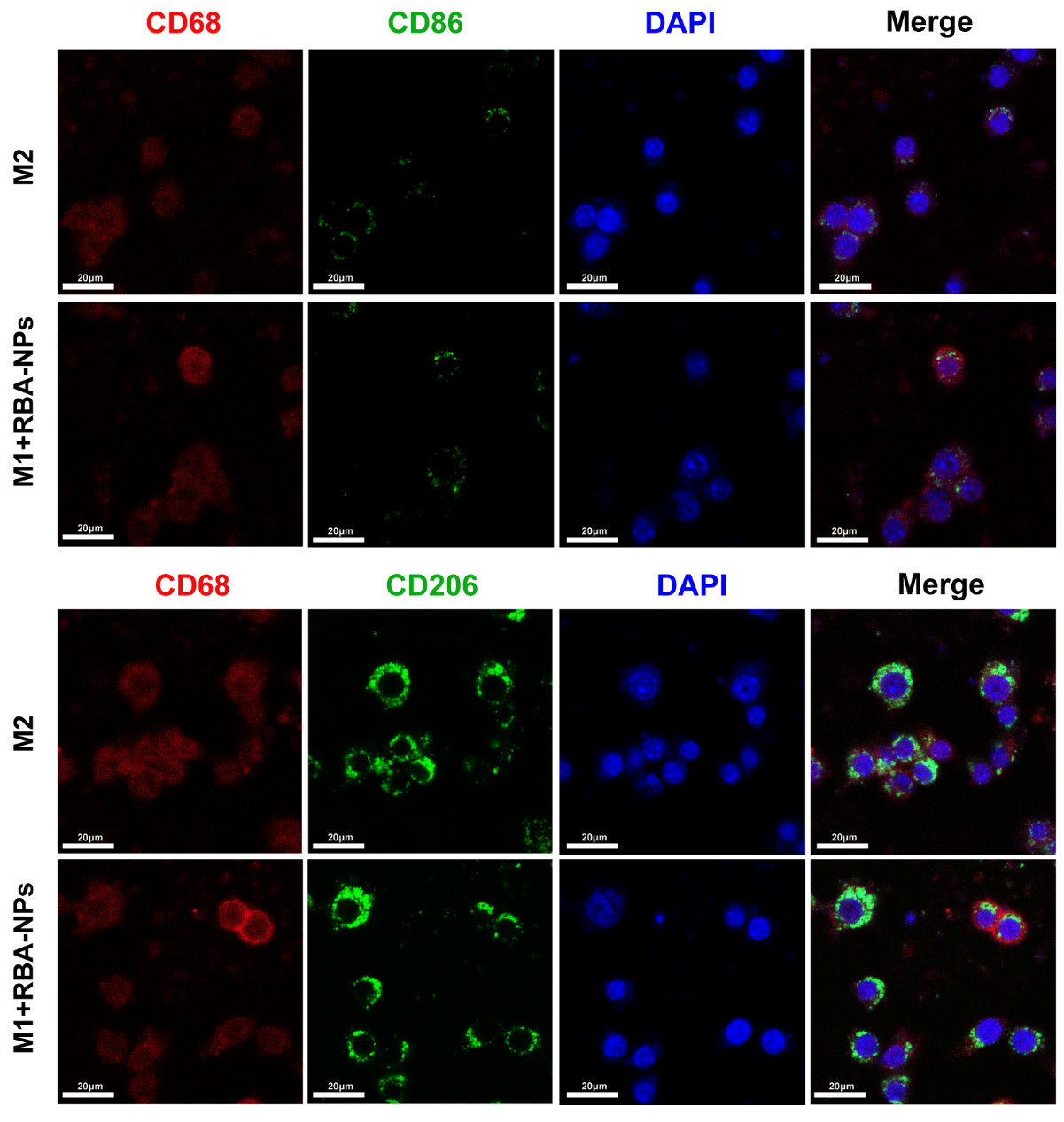


**Figure. S14.** RBA-NPs did not appear to affect the proportion of M2 phenotype macrophages in after IL-4+IL-13 induced THP-1 cells. Immunofluorescence staining of CD68 (red), CD86 and CD206 (green), and nuclei (blue) without or with treatment RBA-NPs. Scale bar = 20 μm.

Figure. S15.


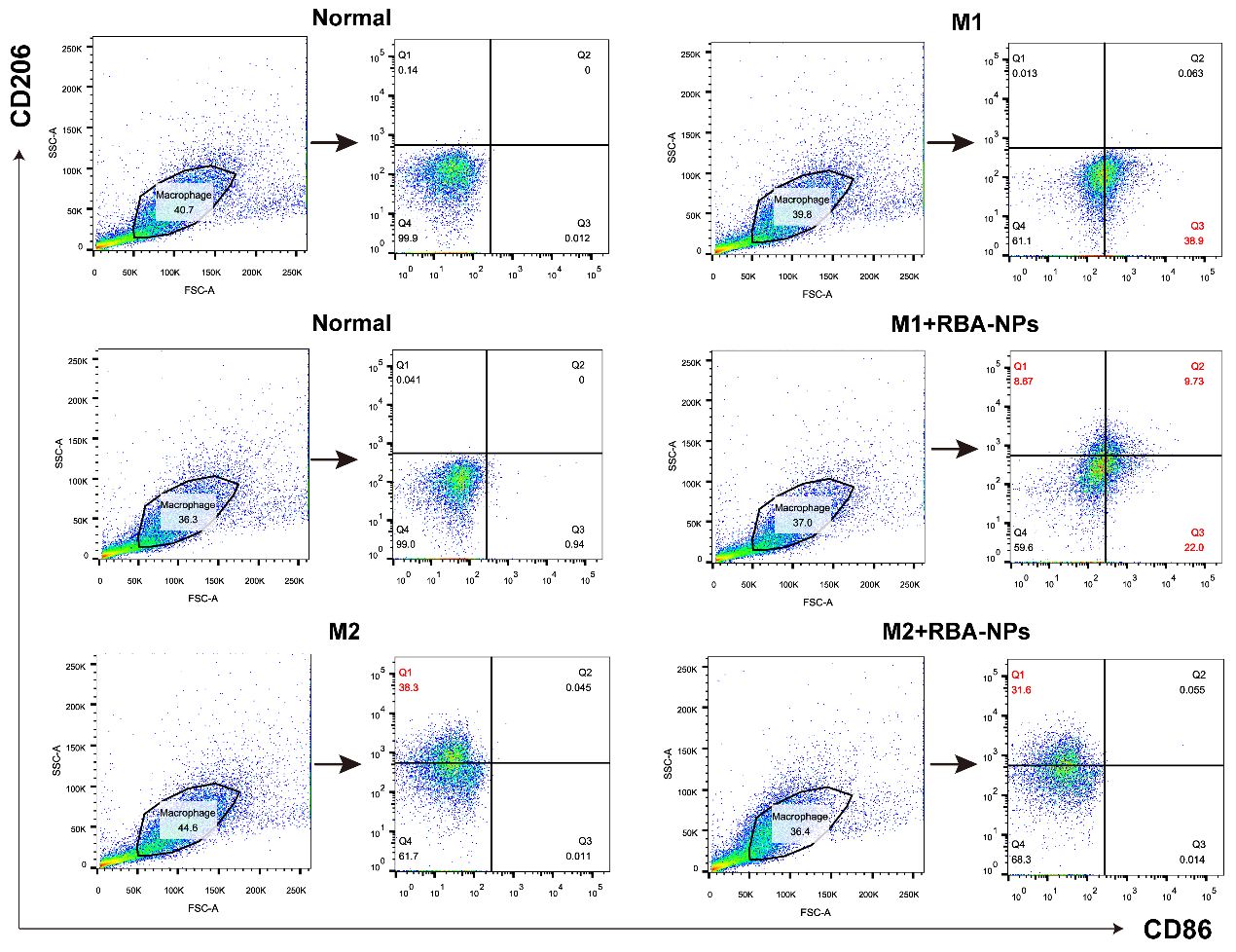


**Figure. S15.** The gate setting schematic for M1 and M2 polarization by flow cytometry in RAW264.7 cells of Figure 5b.

Figure. S16.


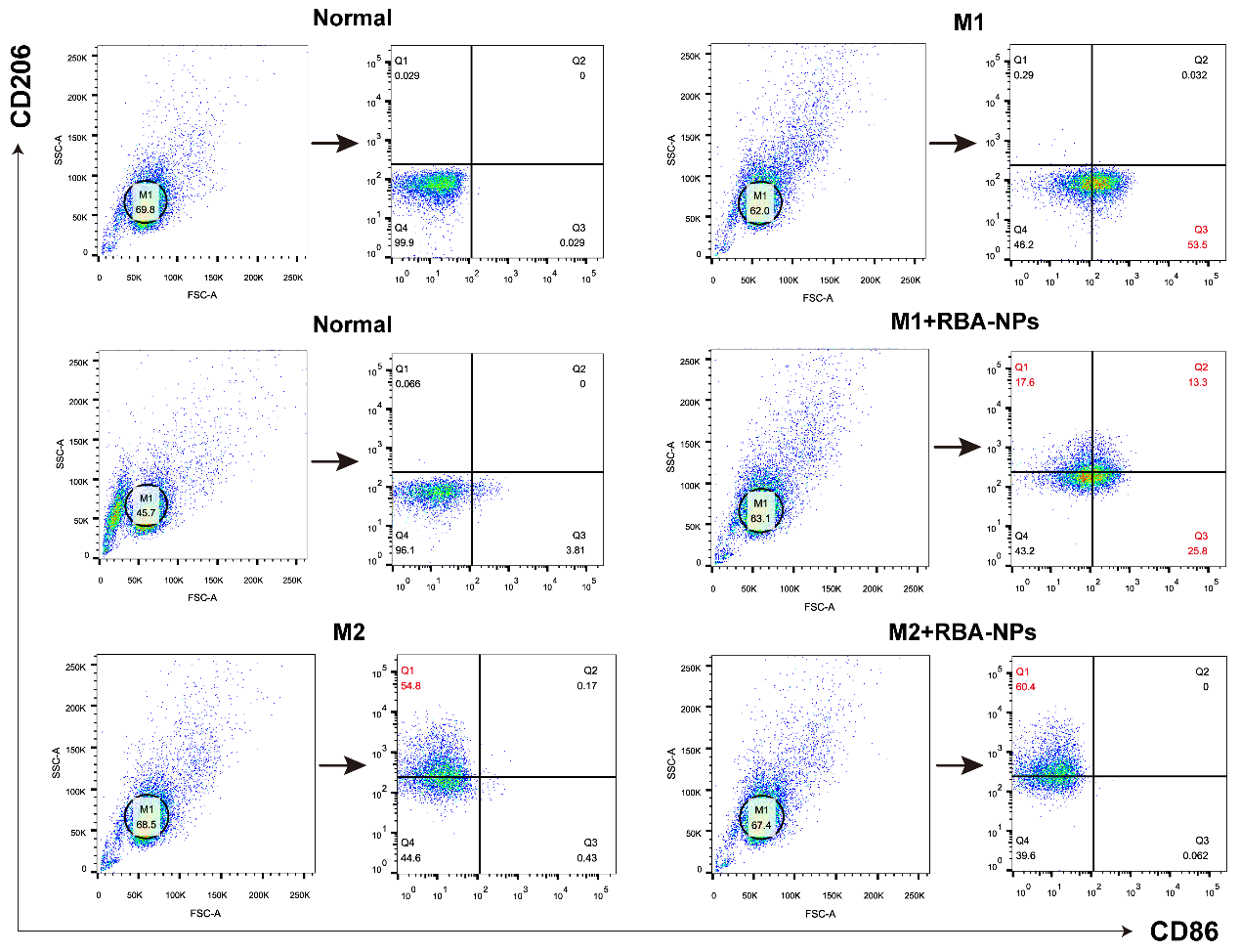


**Figure. S16.** The gate setting schematic for M1 and M2 polarization by flow cytometry in THP-1 cells of Figure 5b.

Figure. S17.


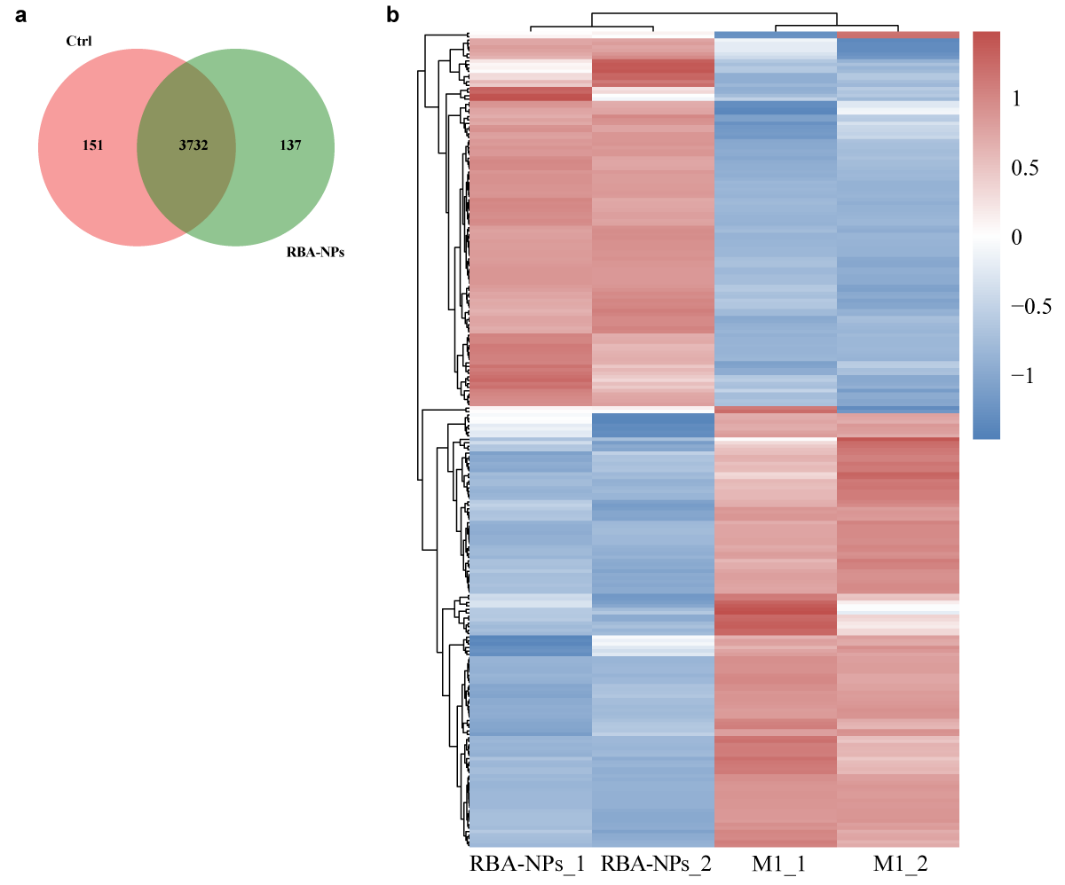


**Figure. S17.** Proteomics analysis of RBA-NPs treated groups compared with control group in M1 macrophages. (a) Venn grams of the whole numbers of differential proteins quantified in proteomics from different groups. (b) Heat map of identified proteins based on hierarchical cluster analysis. Red represents higher upregulation (n = 106) and blue represents higher downregulation (n = 129).

Figure. S18.


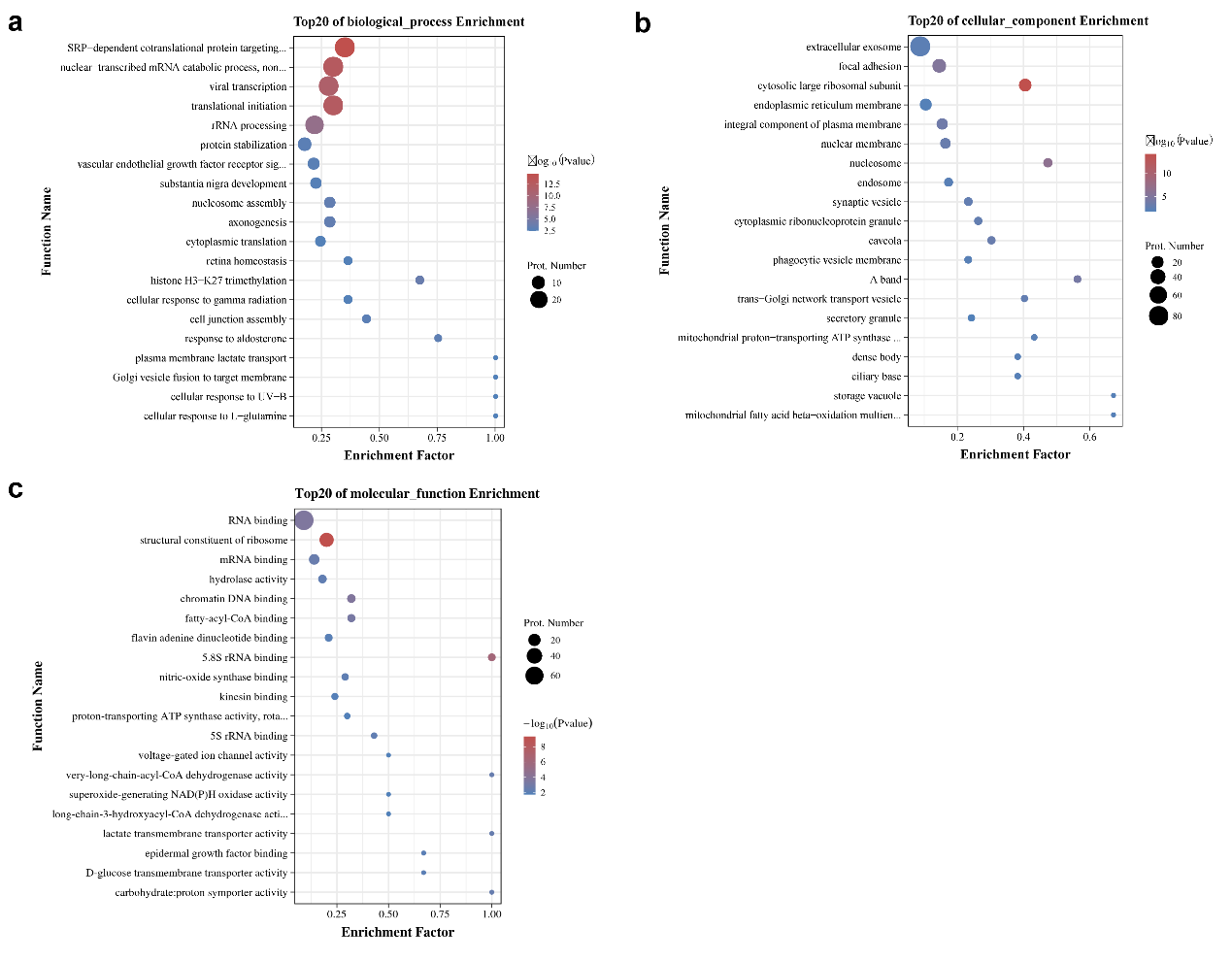


**Figure. S18.** Proteomics analysis of RBA-NPs treated groups compared with control group in M1 macrophages. (a) Venn grams of the whole numbers of differential proteins quantified in proteomics from different groups. (b-d) Top 20 items in the Gene Ontology (GO): (a) biological process (BP), (b) cellular component (CC) and (c) molecular function (MF) enrichment of differential proteins in the proteomic results.

Figure. S19.


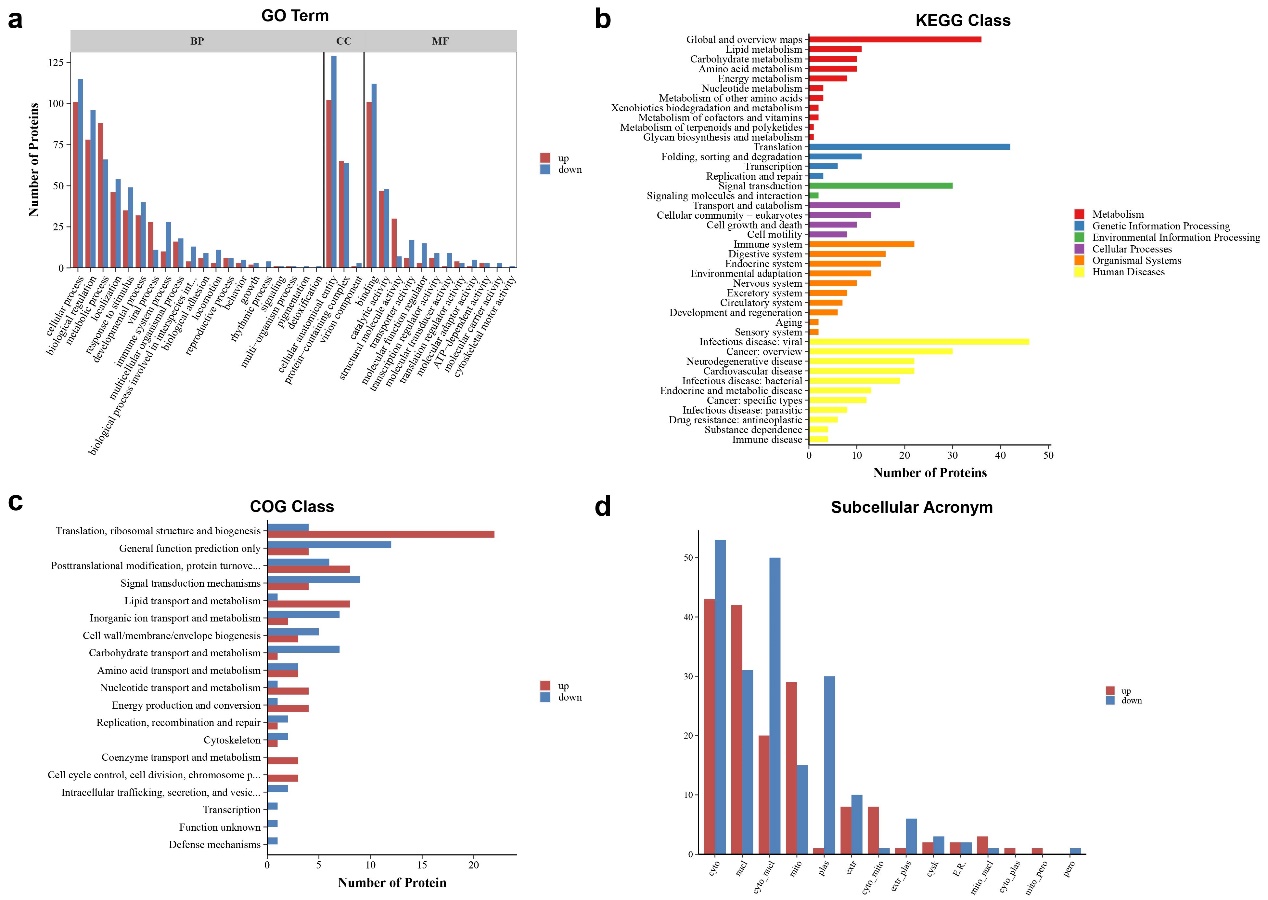


**Figure. S19.** Statistical histogram of annotation results of differential protein GO term (a), KEGG class (b), COG class (c) and subcellular acronym (d).

Figure. S20.


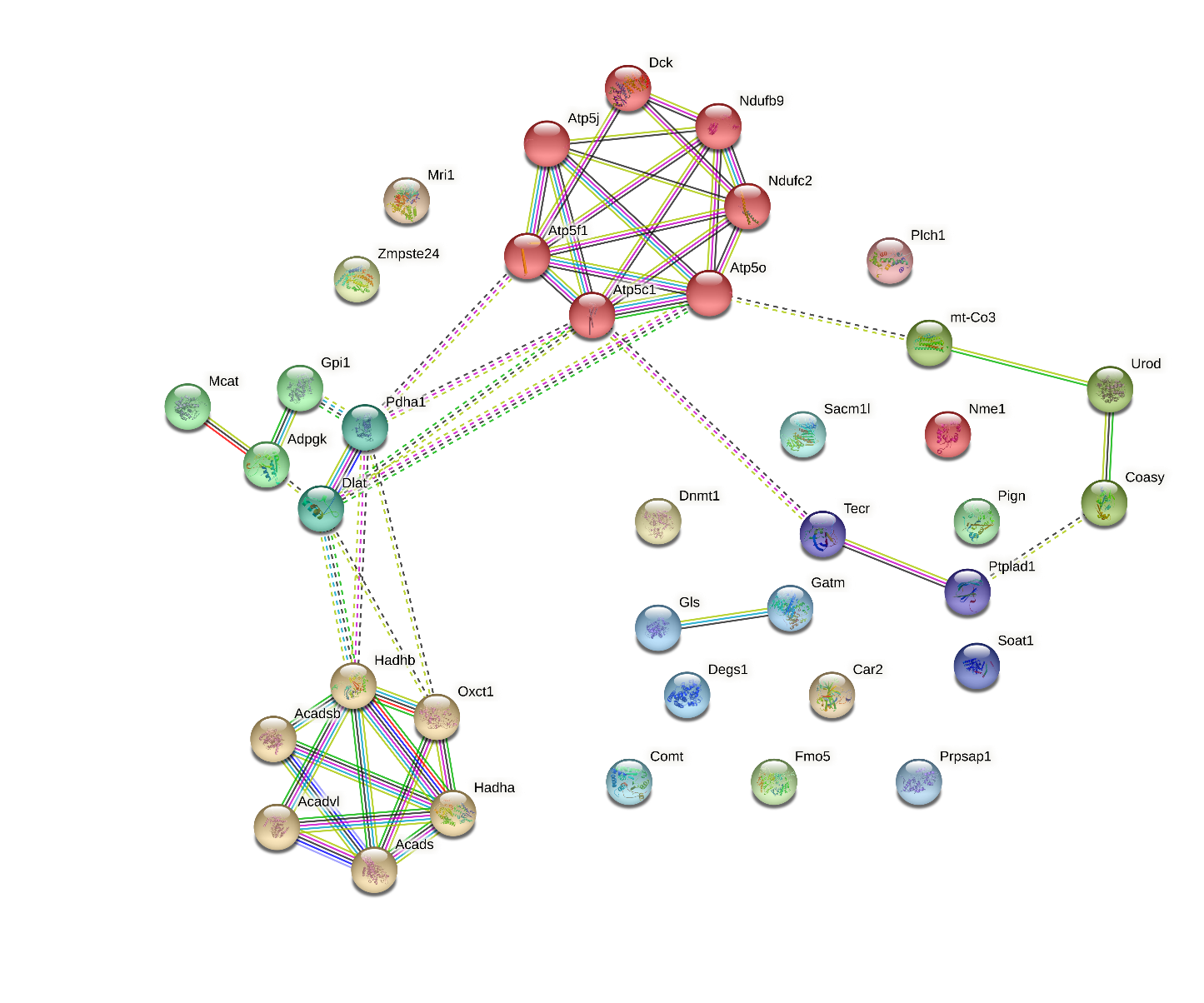


**Figure. S20.** Protein-protein interaction networks to analyze the functional relationships of the 38 proteins constructed with STRING database.

Figure. S21.


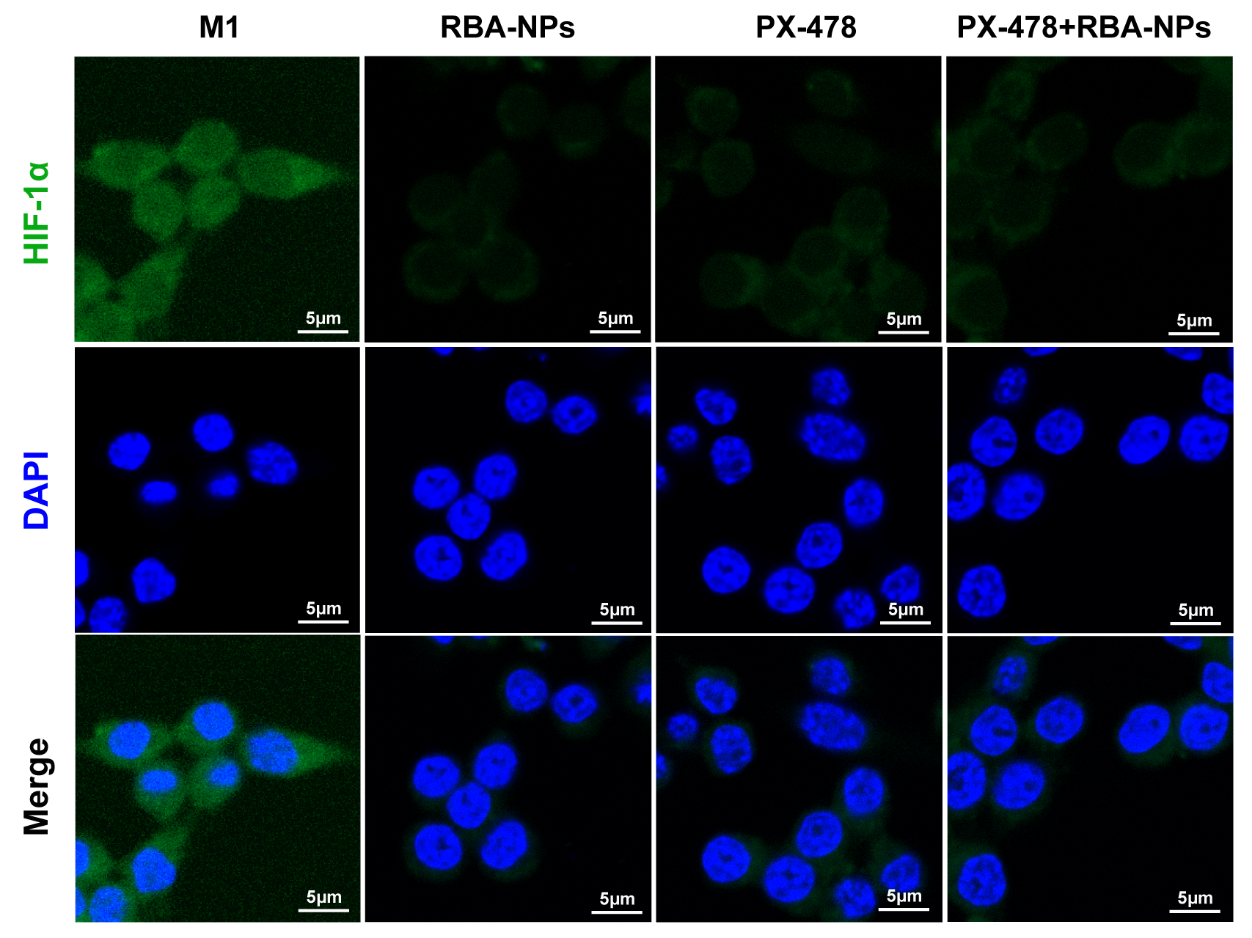


**Figure. S21.** Immunofluorescence staining of HIF-1α (green) and nuclei (blue) on M1 macrophages without or with treatment RBA-NPs or PX-478. Scale bar = 5 μm.

Figure. S22.


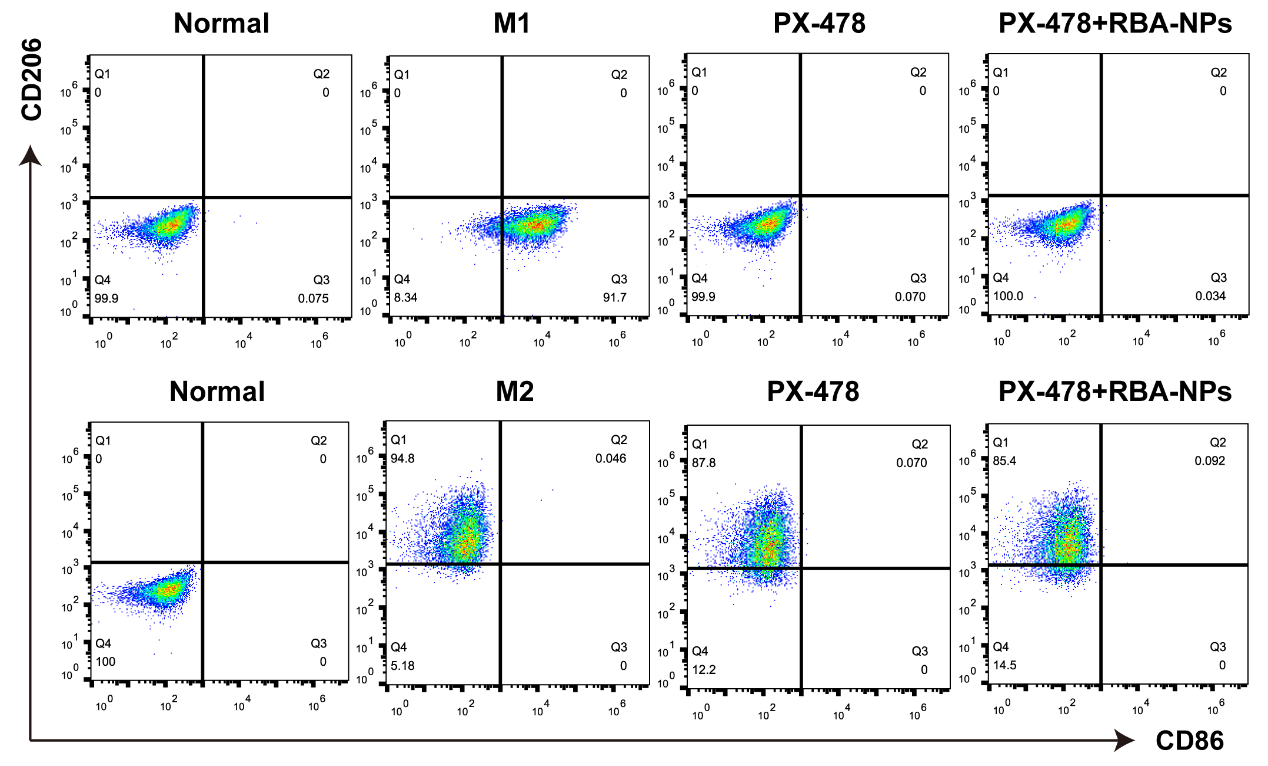


**Figure. S22.** The proportions of M1 phenotype macrophages (CD86+) and M2 phenotype macrophages (CD206+) without or with treatment RBA-NPs or PX-478 were detected by flow cytometry assay.

Figure. S23.


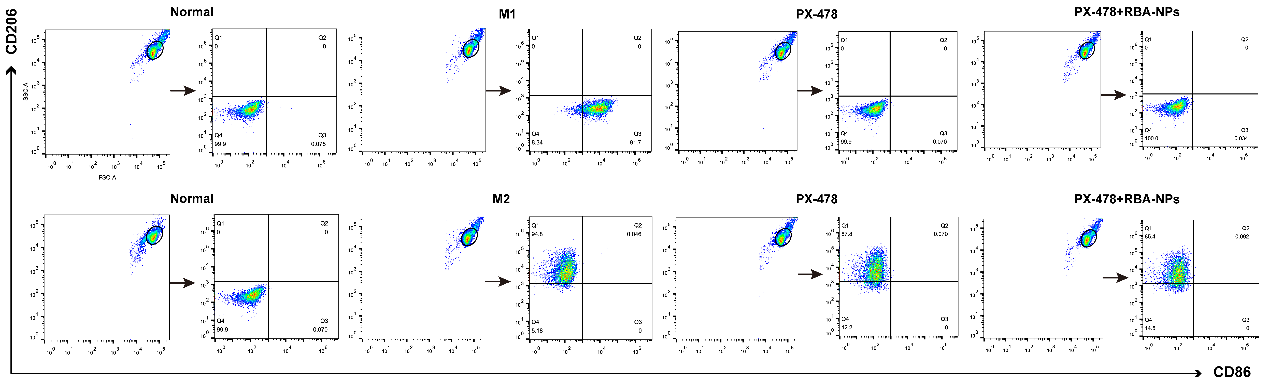


**Figure. S23.** The gate setting schematic for M1 and M2 polarization by flow cytometry of Figure. S22.

Figure. S24.


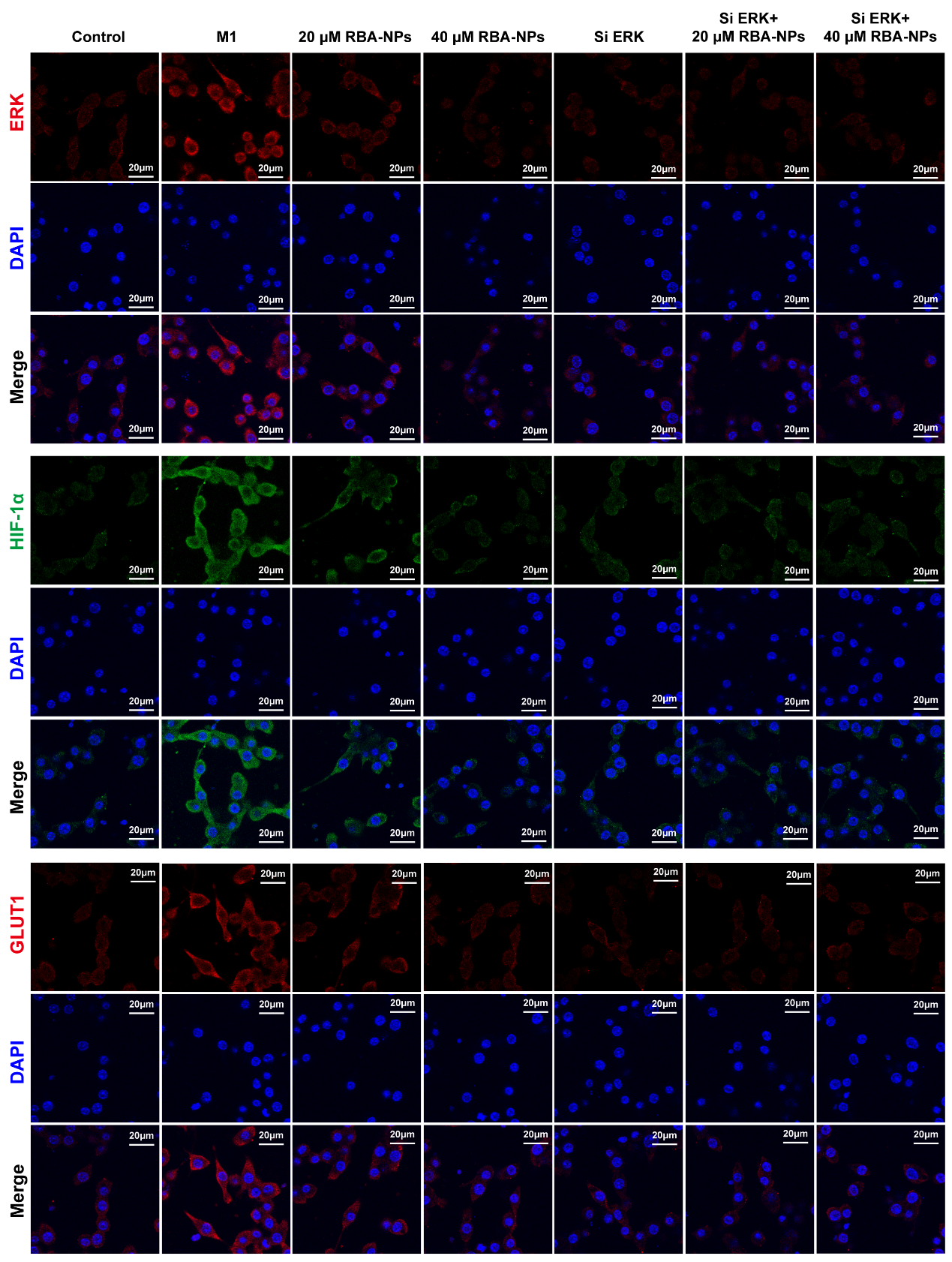


**Figure. S24.** Immunofluorescence staining of ERK (red), HIF-1α (green) and GLUT1 (red) on M1 macrophages in differnent groups. nuclei was stained by DAPI (blue). Scale bar = 20 μm.

Figure. S25.


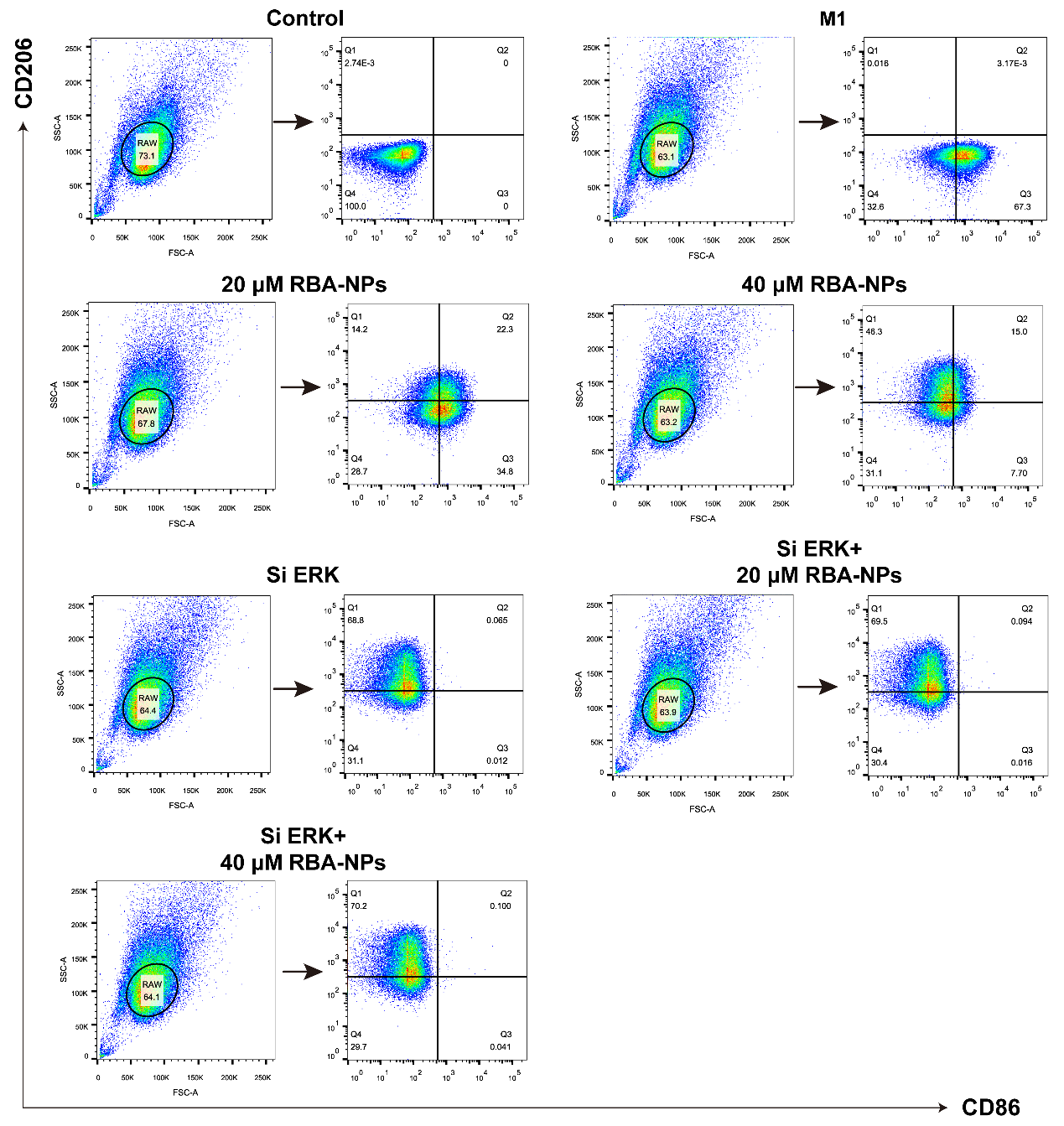


**Figure. S25.** The gate setting schematic for M1 and M2 polarization by flow cytometry of Figure 6k.

Figure. S26.


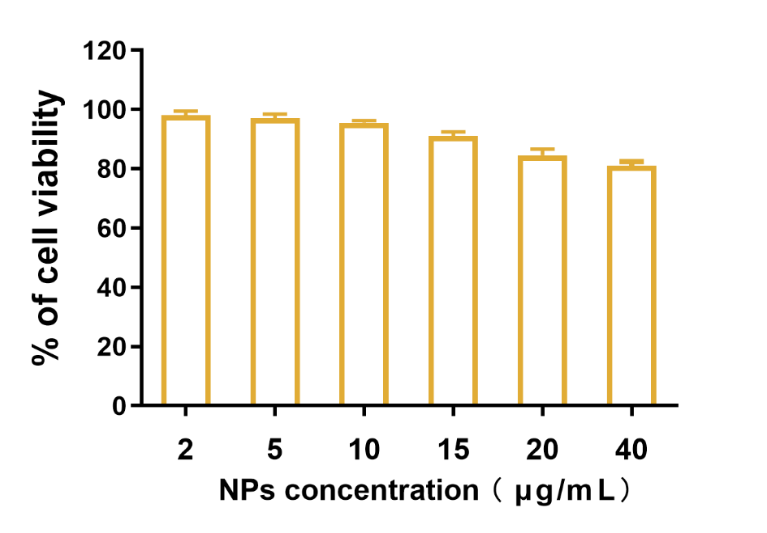


**Figure. S26.** Cytotoxicity of NPs on RAW264.7 cells.

Figure. S27.


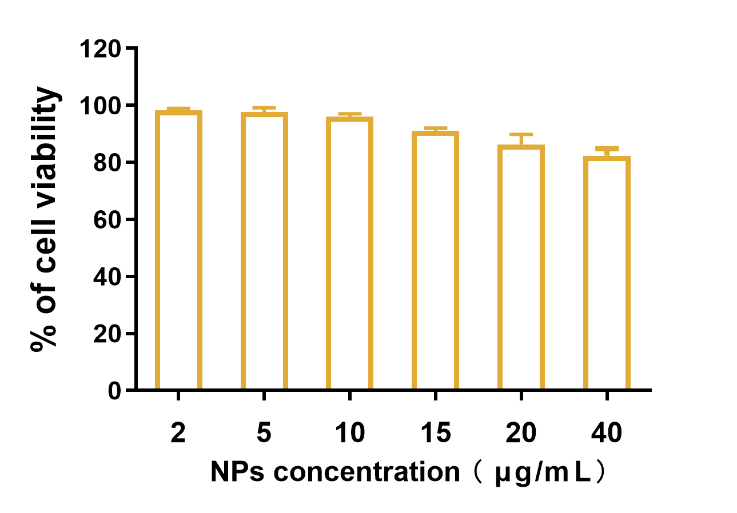


**Figure. S27.** Cytotoxicity of NPs on THP-1 cells.

Figure. S28.


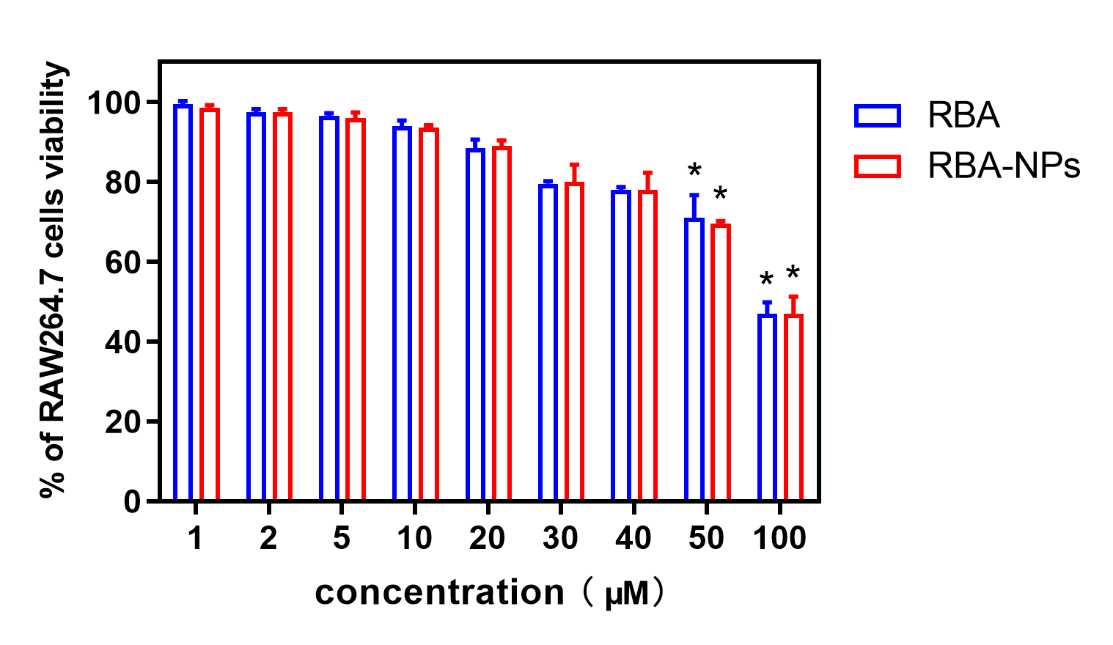


**Figure. S28.** Cell viability of free RBA and RBA-NMs in RAW264.7 cells. Cell viability percent was calculated relative to that of untreated cells (100%). Data shown are mean ± SD.

Figure. S29.


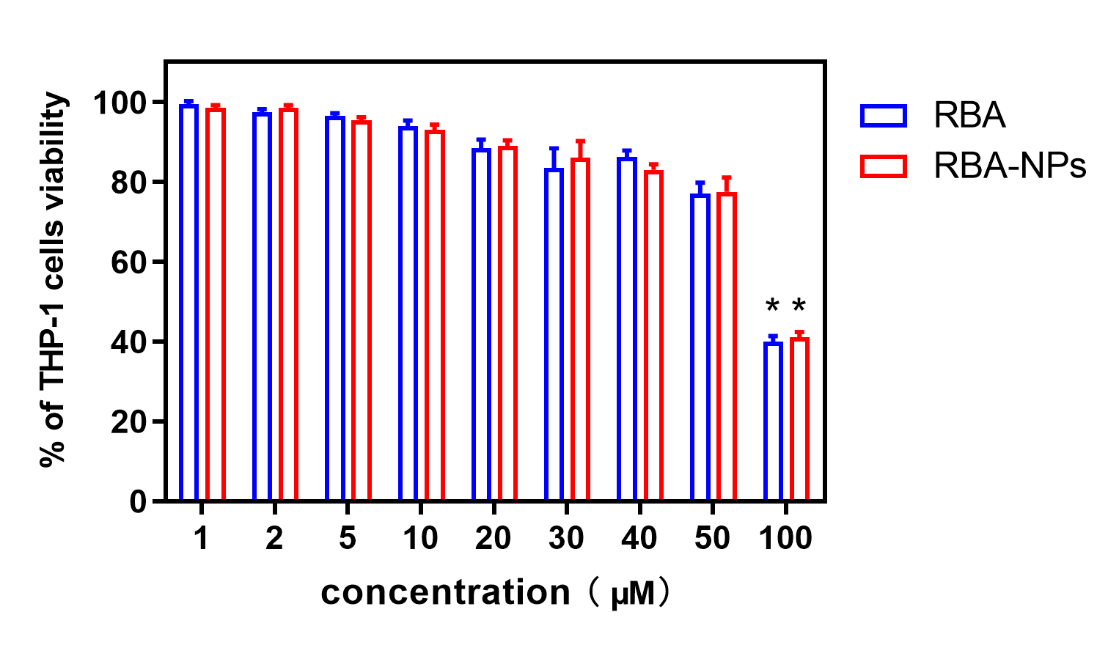


**Figure. S29.** Cell viability of free RBA and RBA-NMs in THP-1 cells. Cell viability percent was calculated relative to that of untreated cells (100%). Data shown are mean ± SD.

Figure. S30.


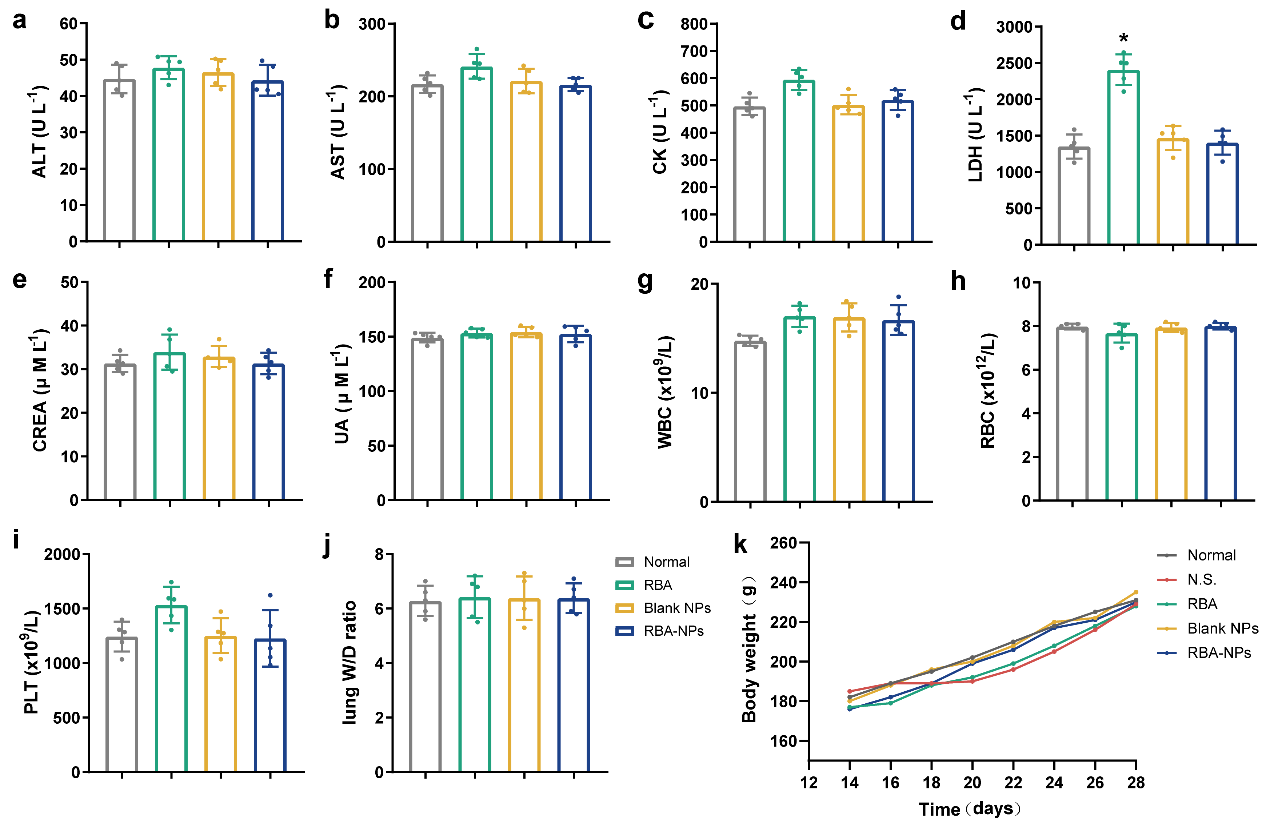


**Figure. S30.** The serum level of ALT (a), AST (b), CK (c), LDH (d), CREA (e), UA (f), WBC (g), RBC (h), PLT (i), lung W/D weight ratio (j) and body weight (k) in rats receiving the indicated treatment. Data represent mean ± SD (n = 5 independent animals). Statistical significance was determined by two-sided Student’s t-test. ALT, alanine aminotransferase; AST, aspartate aminotransferase; CK, creatine kinase; LDH, lactic dehydrogenase; CDEA, creatinine; UA, uric acid; WBC, white blood cell; RBC, red blood cell; PLT, platelet; lung W/D weight ratio, lung wet weight/dry weight ratio.

Figure. S31.


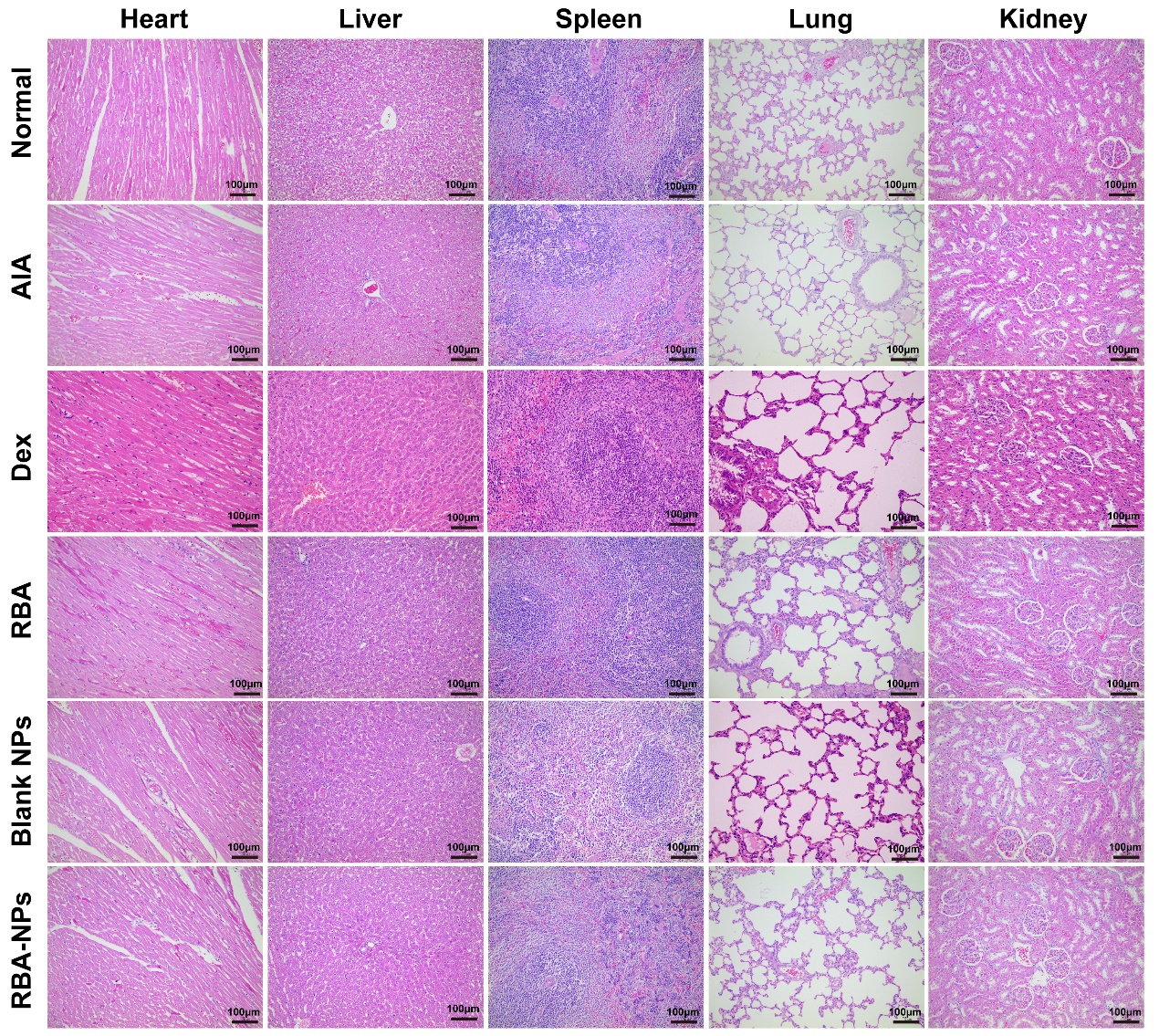


**Figure. S31.** Hematoxylin and eosin (H&E) staining of the major organs (heart, liver, spleen, lung, kidney and brain) were processed 2 days after rats receiving the indicated treatment. (Scale bar = 100 μm) (n = 5 independent animals).

Table S1.

| C (mg/mL) | C _measured_ (mg/mL) | RSD (%) |
| --- | --- | --- |
| 3.125 | 3.04±0.03 | 0.86 |
| 50 | 51.63±1.09 | 2.11 |
| 100 | 96.97±1.68 | 1.73 |

**Table S1.** The precision measurement of FA-HA-PAE .

Table S2.

| C (μg/mL) | Intra-day |  |  | Inter-day |  |
| --- | --- | --- | --- | --- | --- |
|  | C _measured_ | RSD (%) |  | C _measured_ | RSD (%) |
|  | (μg/mL) |  |  | (μg/mL) |  |
| 3.6 | 3.57±0.09 | 2.78 |  | 3.57±0.07 | 1.99 |
| 14.4 | 14.57±0.45 | 3.08 |  | 14.70±0.56 | 3.82 |
| 45.0 | 45.57±0.0.63 | 1.38 |  | 45.55±1.06 | 2.32 |

**Table S2.** The precision measurement of RBA.

Table S3.

| C (mg/mL) | C _measured_  (mg/mL ) | Recovery (%) | Average  Recovery (%) | RSD (%) |
| --- | --- | --- | --- | --- |
|  | 3.06 | 97.98 |  |  |
|  | 3.06 | 97.76 |  |  |
| 3.125 | 3.06 | 97.91 | 97.30 | 0.95 |
|  | 3.03 | 97.08 |  |  |
|  | 2.99 | 95.79 |  |  |
|  | 49.85 | 99.69 |  |  |
|  | 51.71 | 103.43 |  |  |
| 50 | 49.86 | 99.72 | 102.06 | 2.12 |
|  | 51.99 | 103.99 |  |  |
|  | 51.74 | 103.48 |  |  |
|  | 95.13 | 95.13 |  |  |
|  | 97.67 | 97.67 |  |  |
| 100 | 97.70 | 97.70 | 96.19 | 1.42 |
|  | 95.14 | 95.14 |  |  |
|  | 95.31 | 95.31 |  |  |

**Table S3.** The recovery measurement of FA-HA-PAE.

Table S4.

|  | C _measured_ | Recovery (%) | Average | RSD (%) |
| --- | --- | --- | --- | --- |
| C (μg/mL) | (μg/mL ) |  | Recovery (%) |  |
|  | 3.72 | 103.33 |  |  |
| 3.6 | 3.61 | 100.28 | 100.93 | 1.75 |
|  | 3.57 | 99.17 |  |  |
|  | 14.35 | 99.65 |  |  |
| 14.4 | 14.46 | 100.42 | 99.91 | 0.36 |
|  | 14.35 | 99.65 |  |  |
|  | 44.78 | 99.51 |  |  |
| 45.0 | 44.15 | 98.11 | 100.34 | 2.23 |
|  | 46.53 | 103.4 |  |  |

**Table S4.** The recovery measurement of RBA.

Table S5.

| **Protein IDs** | **Protein Name** | **Gene**  **Symbol** | **Mol. weight [kDa]** | **P-Value** | **State** |
| --- | --- | --- | --- | --- | --- |
| tr\|Q3U2J2\|Q3U2J2_MOUSE | Glucose transporter type 1（GLUT1） | Slc2a1 | 54.07 | 5.65231E-06 | down |
| tr\|Q3UF82\|Q3UF82_MOUSE | Mitogen-activated protein kinase（MAPK） | Mapk1 | 40.652 | 2.04611E-05 | down |
| tr\|Q8C6S5\|Q8C6S5_MOUSE | FAD-binding FR-type domain-containing protein | Cybb | 65.309 | 0.000215999 | down |
| tr\|Q0P688\|Q0P688_MOUSE | Eukaryotic translation initiation factor 4E member 2（EIF4E2） | Eif4e2 | 27.66 | 0.023457442 | down |

**Table S5.** Differential proteins enriched in HIF-1 signaling pathway (ko04066).
